# Nanotechnology-enabled immunoengineering approaches to advance therapeutic applications

**DOI:** 10.1186/s40580-022-00310-0

**Published:** 2022-04-28

**Authors:** Skylar T. Chuang, Brandon Conklin, Joshua B. Stein, George Pan, Ki-Bum Lee

**Affiliations:** Department of Chemistry and Chemical Biology, Rutgers, The State University of New Jersey, Piscataway, NJ 08854 USA

**Keywords:** Nano-immunoengineering, Immunotherapy, Nanoparticles, Cancer, Vaccines, CAR T-cell therapy, Tolerance, Tissue regeneration, Gene delivery

## Abstract

Immunotherapy has reached clinical success in the last decade, with the emergence of new and effective treatments such as checkpoint blockade therapy and CAR T-cell therapy that have drastically improved patient outcomes. Still, these therapies can be improved to limit off-target effects, mitigate systemic toxicities, and increase overall efficacies. Nanoscale engineering offers strategies that enable researchers to attain these goals through the manipulation of immune cell functions, such as enhancing immunity against cancers and pathogens, controlling the site of immune response, and promoting tolerance via the delivery of small molecule drugs or biologics. By tuning the properties of the nanomaterials, such as size, shape, charge, and surface chemistry, different types of immune cells can be targeted and engineered, such as dendritic cells for immunization, or T cells for promoting adaptive immunity. Researchers have come to better understand the critical role the immune system plays in the progression of pathologies besides cancer, and developing nanoengineering approaches that seek to harness the potential of immune cell activities can lead to favorable outcomes for the treatment of injuries and diseases.

## Introduction

The immune system is an inevitable part of the human body, interfacing with every organ system. Primary immune functions are to maintain homeostasis; insufficient or excess immune response can lead to pathologies such as cancer, infectious disease, chronic inflammation, and more. Recent advances in biomedical and pharmaceutical engineering have allowed researchers to engineer immune cells to further our understanding of the complex processes and develop better treatments. Moreover, since many biomedical processes (drug release, cellular uptake, signal transduction) occur on the nanoscale, controlling the immune system by nanoscale engineering, or nano-immunoengineering, can lead to more favorable outcomes.

Herein, we define the concept of nano-immunoengineering as engineering approaches that seek to enhance, control, or regulate immune cell functions by incorporating nanoengineering concepts and designs. The knowledge accumulated from these results will be used to design better treatments for various biomedical applications. These include, but are not limited to, designing better synthetic and biomaterials for delivering immunomodulatory drugs or biologics [1], increasing drug and gene delivery efficiency in target immune cells [2], and enhancing immune cell behavior with nanomaterials [3]. Being that this is a new field that is rapidly expanding, with growing interest in public institutions, private sectors and funding bodies, the translational impact of nano-immunoengineering is expected to increase even more in the next decade [4].

**Fig. 1:**
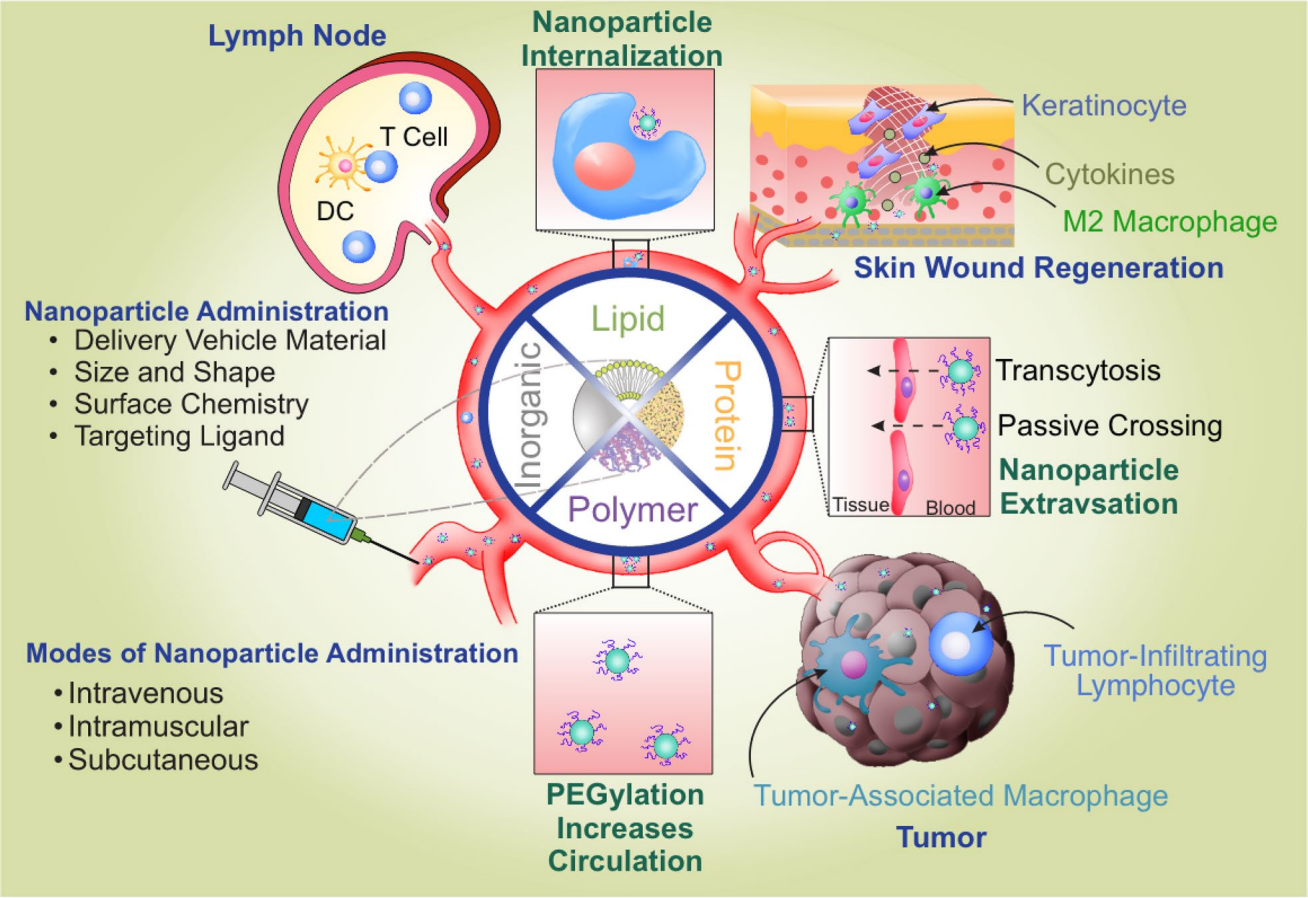
Overview of nano-immunoengineering designs and applications. Four major types of materials suited for biomedical applications include lipids, polymers, proteins, and inorganic materials. Therapeutic cargoes encapsulated in lipid coatings or grafted with poly (ethylene glycol) (PEG) can increase stability and circulation half-life. Nanoparticles can extravasate into tumors or tissues depending on the size, shape, charge, and hydrophobicity. In the tumors, the nanoparticles could enhance T cell functions or reprogram tumor-associated immune cells to improve anti-tumor efficacy; in the tissues, nanoparticles engulfed by dendritic cells (DCs) could either promote host immunity or tolerance when they migrate to the lymph nodes; in injured tissues, nanoparticles could reprogram resident immune cells such as macrophages to a healing phenotype to accelerate wound healing

In this Review, we discuss the different aspects of nano-immunoengineering, emphasizing zero-dimension nanomaterial platforms that could modulate the immune system to induce an immune response against cancers and pathogens, or promote tolerance and tissue regeneration (Fig. 1). We will place a heavy emphasis on nanoparticle platforms because the site of action predominantly occurs in the cardiovascular and lymphatic system or deep tissues where nanoparticles could penetrate and accumulate more efficiently. This is in contrast to other two-dimension or three-dimension nanomaterials such as nanofibers, nanowires, nanosheets and nanoscaffolds, where their applications are better suited for topical or surgical procedures. We discuss the implication of these platforms and highlight the cutting-edge technologies that have the potential to revolutionize the field of immunotherapy.

## Enhancing anti-tumor immunity for cancer immunotherapy

**Fig. 2:**
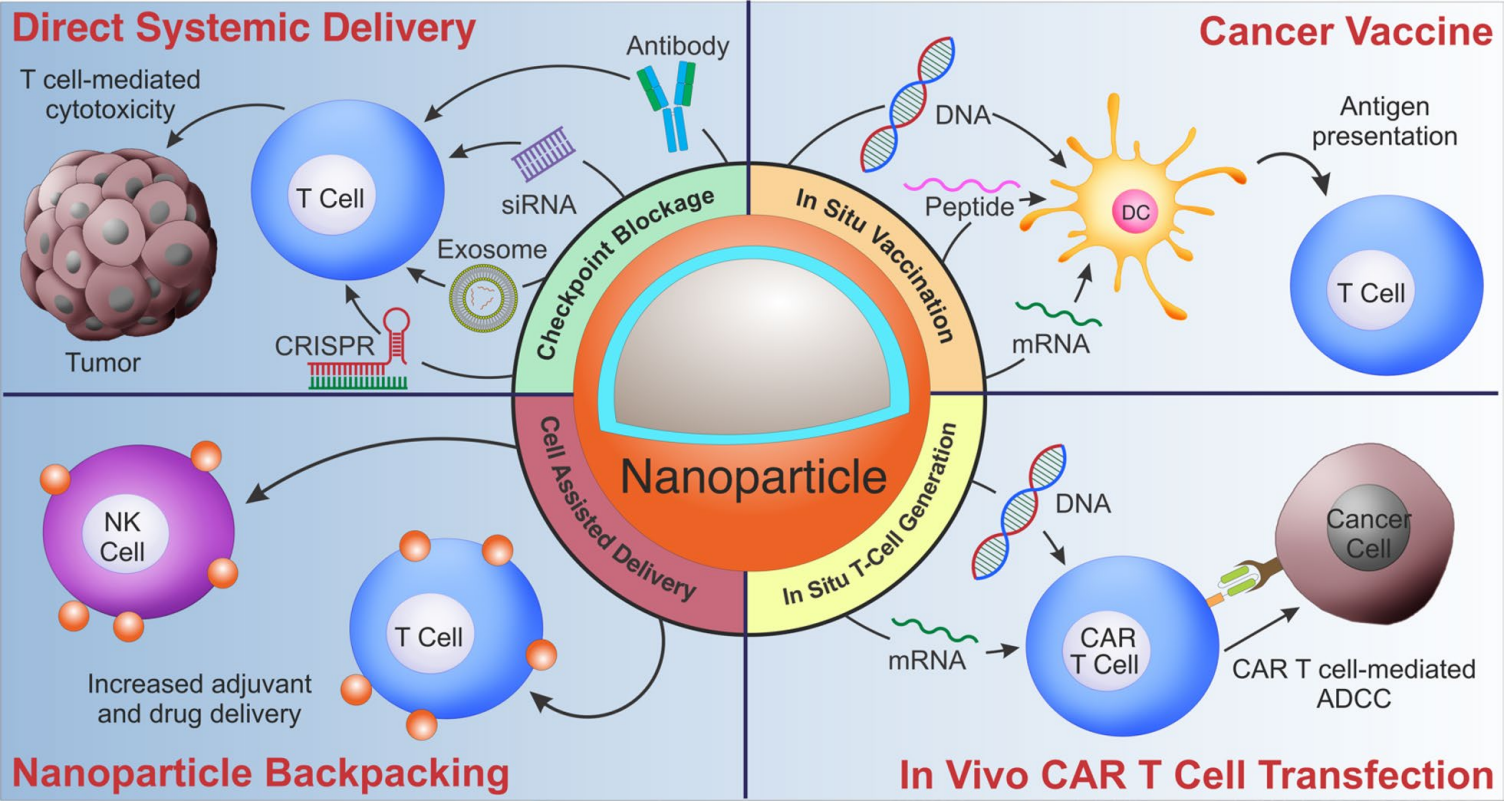
Nano-immunoengineering for cancer therapy. Nanoparticles can be utilized for various applications, including (i) delivering immunomodulatory molecules, including checkpoint inhibitors in the form of proteins or nucleic acids to enhance T cell immunity or reprogram the tumor microenvironment; (ii) generating effective cancer vaccines via transfecting DCs; (iii) attaching nanoparticles to T cells and NK cells to maximize localized delivery of adjuvant or drugs and limit systemic toxicities; iv) producing CAR T-cells in situ by transfecting endogenous T cells

One of the most prominent applications of nano-immunoengineering is in combating cancer. The advent of immunotherapy, combined with next-generation sequencing and systems biology, allows scientists and clinicians to develop therapies for precision oncology. Still, there is a gap between translating such information into therapies; this includes developing carriers that can maximize bioactivity and bioavailability and enhance target immune cell functions through such properties. In this section, we introduce applications of nano-immunoengineering in the delivery of immunomodulatory agents and cancer vaccines, as well as enhancing and modulating adoptive cell therapy (ACT) (Fig. 2).

### Nanotechnology for effective delivery of immunomodulatory agents

Nanotechnology offers several advantages in the delivery of therapeutic cargoes for modulating the activity of immune cells. This includes more selective targeting, controlled release of payload, greater biodistribution, and prolonged and enhanced effects of the immunomodulator via the design of the nanoparticle (surface charge, size, hydrophobicity, stiffness, pore size, biocompatibility, and biodegradability). In addition to directly enhancing the anti-tumor immunity of immune cells, such as T cells or NK cells, “re-educating” tumor-associated immune cells such as tumor-associated macrophages (TAMs) represents a promising strategy for immunotherapy and immunoengineering. As macrophages are highly plastic cells, they can polarize to the pro-inflammatory, tumoricidal M1 phenotype, or the anti-inflammatory, pro-tumorigenic M2 phenotype. In particular, TAMs, classified as M2d, represent a critical therapeutic target since they constitute the major immune cell populations in tumors and mediate immunosuppression via the secretion of IL-10 and TGF-β and coordinate with other cells such as myeloid-derived suppressor cells (MDSCs) and regulatory T cells (T_reg_) [5]. Strategies that seek to deplete [6] or re-polarize [7] TAMs represent have been heavily investigated, and this tumor microenvironment (TME) reprogramming approach represents a promising therapeutic strategy for cancer immunotherapy by virtue of enhancing anti-tumor immunity. Herein, we provide a brief overview of how nanotechnology can be combined with immunomodulatory agents, focusing specially on checkpoint blockade and microenvironment reprogramming; a more extensive review of checkpoint inhibitors and their application in nanomedicine can be found elsewhere [8].

#### Delivery of protein-based immunomodulators

A particular field that has significantly advanced in the last decade is the development of checkpoint inhibitors, mostly in the form of monoclonal antibodies (mAbs). Especially, programmed cell death protein 1 (PD-1) is a major immune checkpoint expressed by activated T cells, B cells, and monocytes. The engagement of PD-1 and its ligand, PD-L1, elicits an inhibitory signal in activated T cells; many cancers hijack this pathway to evade immune surveillance. Blocking this interaction using PD-1 antibodies can overcome this immunosuppression and enhance anti-tumor immunity. While mAb-based checkpoint blockade therapy has tremendous clinical success, systemic administration can still suffer lower efficacy due to premature degradation of the antibody and off-target toxicities. Several groups have worked on the direct delivery of PD-1 antibodies using biodegradable nanocarriers like PLGA [9, 10] or large pore mesoporous silica-upconversion nanoparticles (UCNP) [11]. PD-1 antibodies can be co-delivered with other adjuvants or small molecule inhibitors to improve efficacy. For translational use, efforts focusing on developing biodegradable platforms will allow more excellent safety and release of the encapsulated PD-1 antibodies. Recent advances have focused on triggered-release systems based on tumor microenvironment cues, such as pH, reactive oxygen species (ROS), or matrix metalloproteinase (MMP) expression. The expression level of MMP has been correlated with tumor metastasis, making such biological cues an excellent target. A MMP-mediated, biodegradable DNA nano-cocoon has been developed in which both CpG-oligodeoxynucleotides (CpG-ODN) and PD-1 antibodies could be co-loaded into the same nanocomplex for preventing postsurgical tumor relapse [12]. The release of the cargoes is mediated by the cleavage of the triglycerol monostearate capsules encasing the restriction enzyme Hhal by MMPs. When freed, the Hhal can degrade the nano-cocoon via restriction digest, thereby releasing CpG-ODN and the encapsulated PD-1 antibodies. The CpG DNA herein not only acted as a delivery vehicle, but also as a therapeutic agent that could enhance the anti-tumor response in a B16F10 metastasis mouse model. In a separate study utilizing MMP2-mediated degradation, Liu et al. co-encapsulated IMD-0354-containing lipid nanoparticles (~ 32 nm) with PD-1 antibodies in a nanogel (final size ~ 120 nm) to achieve PD-1 blockade and TAM repolarization. IMD-0354 is a NF-Kβ pathway inhibitor that can downregulate PD-1 expression on the surface of activated T cells. The combination approach allowed targeting both T cells and M2 TAMs, leading to significant tumor growth inhibition and extended survival of mice bearing B16 tumors. Besides PD-1 antibodies, other checkpoint inhibitors have been directly delivered or combined with other nanomaterials to achieve greater therapeutic efficacy, including CTLA-4 [13, 14] and CD47 [15].

#### Delivery of nucleic acid-based immunomodulators

Another promising approach to modulate the immune system is by delivering nucleic acids such as plasmid DNA (pDNA), messenger RNA (mRNA), short interfering RNA (siRNA), and microRNA. In contrast to mAbs, whose microheterogeneity patterns can influence their characteristics and encapsulation efficiency [16], the negative charge associated with nucleic acids allows them to be readily encapsulated using cationic materials with high efficiency. In particular, siRNA-mediated knockdown has been formulated into various types of nanoparticles composed of lipids [17], polymers [18], and inorganic matrices [19] for modulating the target immune cell. A significant advantage of RNA therapeutics over DNA therapeutics for immunotherapy is that RNA can function readily in the cytosol, whereas DNA must localize to the nucleus for proper expression. This generates a major barrier for nonviral plasmid DNA delivery into immune cells, as many of them, including primary T cells, NK cells, DCs, and macrophages, are refractory to transfection with pDNA using chemical-based methods. Therefore, major work in nanoparticle-based nucleic acid delivery has focused on RNA over DNA as the therapeutic cargo for immunomodulation. Recent efforts have focused on developing biodegradable platforms for clinical translation, like protein therapeutics. These biodegradable platforms could consist of either polymeric, lipid, or inorganic materials. A hybrid lipid calcium phosphate nanoparticle (LCP NPs) was developed that encapsulated PD-1 siRNA [20]. This material was originally developed by the Leaf Huang group for enhanced delivery of nucleic acids due to the synergistic effect of lipid-mediated membrane fusion and the proton sponge effect from the degradation of calcium phosphate in eliciting endosome escape [21]. Furthermore, calcium phosphate has the advantage of being biocompatible and completely biodegradable under acidic pH. These small (~ 30 nm) nanoparticles could readily encapsulate nucleic acids via the electrostatic interaction between Ca^2+^ and the PO_4_^3−^ backbone of DNA or RNA. The delivered siRNA could readily knock-down PD1 expression in tumor-infiltrating lymphocytes, resulting in greater killing efficacy and cytokine production [20]. Compared to tumor-infiltrating lymphocytes, tumor-infiltrating monocytes and macrophages contribute significantly to tumor progression, invasion, and metastases. Hanafy et al. has developed lipid nanoparticles containing acid-labile PEG linkers for the encapsulation of PD-1 siRNA for the downregulation of PD-1 on TAMs as opposed to lymphocytes. They observed increased uptake of the acid-sensitive PEG lipid nanoparticles in J774A.1 macrophages. Notably, both the PD-1 expression in the CD68 + TAMs and tumor size were greatly reduced in a B16-F10 tumor mouse model. The authors attributed the results to the re-polarization of M2 to M1 macrophages upon checkpoint blockade. In addition to PD-1 blockade, knockdown strategies that target key genes involved in the pro-tumorigenic functions of TAM also generated positive outcomes. In a recent study, lipid nanoparticles based on an ionizable lipid CL4H6 were developed for the silencing of activator of transcription 3 (STAT3) and hypoxia-inducible factor 1 α (HIF-1α) in TAMs [17]. STAT3 and HIF-1α are known to interfere with tumor suppression and increase tumor angiogenesis [22, 23]. The nanoparticles were produced using an ethanol dilution method, giving rise to homogenous (PDI 0.0–0.2) and small (~ 90 nm) particles, with a relatively neutral zeta potential with a high encapsulation efficiency (> 90%) of the siRNA. TAMs readily took up the lipid nanoparticles compared to other cell populations (i.e. tumor cells, endothelial cells, and other leukocytes) even in the absence of targeting ligands. Screening using both RAW 264.7 cells (murine macrophages) and bone marrow-derived macrophages (BMDM) showed, as well as TAMs in vivo, showed that a ratio of 60:40 or 70/30 mol% of CL4H6:chol enabled the greatest silencing activity. The inhibition of STAT3 and HIF-1α (by 37% and 48%, respectively) led to the infiltration of CD11 + macrophages as well as an increase in the presence of CD169^+^ (M1) macrophages. In addition, quantitative PCR revealed a decrease in CD31 and TGF-β levels, as well as an increase in IFN-γ and TNF-α levels, accompanied by a significant reduction of tumor size. To enhance the efficacy and targeting of TAMs, researchers have focused on key receptors, such as CD163 and CD206. Of the many receptors, CD206, or the macrophage mannose receptor 1 (MRC1), has been commonly utilized as the target for several nanoparticle delivery systems [24–26]. Zhang et al. synthesized a poly(β-amino ester) (PBAE) nanocarrier coated with poly(glutamic acid)- mannose for the targeted co-delivery of in vitro-transcribed (IVT) IRF5 and IKK mRNA (3:1) in an ovarian cancer mouse model [26]. The nanocarriers successfully reprogrammed the M2 TAMs into an M1 phenotype, slowed the tumor growth, and doubled the survival time in the mice. This platform was further applied to mice with pulmonary melanoma metastases and glioma. However, the authors did not observe complete eradication of the tumors in the various mouse models tested, suggesting that this approach is best used in combination with other therapies for efficacy.

#### Gene editing approach for immunomodulation

The CRISPR-Cas system has emerged as a powerful tool in modulating the immune system. Due to the large loading capacity required for the CRISPR-Cas system, nonviral nanocarriers could enable such delivery in vivo. For instance, Li et al. reported the synthesis of nanoparticles with different PEG densities containing CRISPR-Cas for the in vivo targeting of B cells [27]. However, for therapeutic applications, the delivery of the ribonucleoproteins (RNPs) is preferred to limit off-targeting effects and unwanted gene editing, genome toxicity, and immunogenicity. By using a truncated Cas9 targeting sequence and poly(glutamic acid) (PGA) as an RNP stabilizer, Nguyen et al. showed fourfold improved HDR efficiency in CD4^+^ T cells using this nanoparticle platform combined with electroporation [28]. This enhanced efficiency was also observed in other types of immune cells such as CD8^+^ T cells, B cells, NK cells, and hematopoietic stem cells (HSCs). To bypass the use of electroporation for RNP delivery, the Rotello group developed a nanocomposite platform using engineered RNP with gold nanoparticles. By incorporating the RNP with an oligo(glutamic acid) tag, the protein could readily associate with gold nanoparticles that contained arginine head groups via carboxylate-guanidinium interaction to form nanocomposites that were about 285 nm in size [29]. The relatively large size of these nanocomposites provided a passive targeting strategy for macrophages in vivo. Notably, compared to other nanocarrier systems that were less efficient at endosome escape (hence lower editing efficiency), this approach led to direct cytosolic delivery of the RNPs, and knockout of the PTEN gene in macrophages in vivo was successful. While many current oncology studies have focused more on the delivery of the RNP into tumor cells rather than directly into immune cells, this strategy can still lead to enhanced intratumor immune response via the suppression of the immune checkpoints. In addition, the co-delivery of small molecule drugs can lead to immunogenic cell death, further contributing to anti-tumor immunity. Liu et al. reported a virus-like nanoparticle (VLN) that co-delivered the CRISPR/Cas system along with small molecule drugs for combination therapy [30]. The particle core comprised of thiolated mesoporous silica nanoparticles (MSN) in which the pores were loaded with axitinib, a tyrosine kinase inhibitor that suppresses tumor growth via the MAPK-ERK and P13K-AKT pathway; the pores were “sealed off” by RNP with sgRNA targeting PD-L1 that were conjugated to the surface via disulfide bonds. The VLN core was further coated with a layer of lipids to enhance the stability and particle uptake in tumor cells. This triggered release system could be initiated by intracellular glutathione, upon which the RNP could dissociate, along with the release of axitinib in the target cancer cells. The VLN was able to achieve a knockdown efficiency up to 58.2% and reduced the expression of PD-L1 by up to 41.3% in B16F10 cells. These in vitro results reflected a significant reduction of Treg population and tumor size in vivo.

#### Exosomes for immunomodulation

Besides synthetic nanomaterials, naturally-derived nanoparticles such as exosomes have also been applied for immunomodulation. Exosomes are small (30–150 nm), spherical extracellular vesicles (EVs) generated by cells that contain a variety of biomolecules such as proteins, mRNAs, and microRNAs for cellular communication. Exosomes include tetraspanins such as CD9, CD37, CD63, and CD81, which can be employed as biomarkers to isolate them for biosensing and disease detection. Exosomes have lately acquired popularity in immunotherapy, owing in part to their ease of preparation, storage, and manipulation when compared to ACT. Exosomes harvested from various types of immune cells, such as DCs, NK cells,  CD8^+^T cells and M1-polarized macrophages, have all shown to exert anti-tumor effects or potentiate such responses [31–34]. Notably, the cytotoxic activity of the exosomes is mainly dependent on the cytokines used to activate the immune cells, such as IL-12 for CD8^+^ T cells [35] and IL-15/IL-21 for NK cells [36]. Besides cancer cells within tumors, exosomes derived from CD8^+^ T cells and NK cells have been shown to mediate cytotoxic activity against tumor stromal cells such as mesenchymal stromal cells (MSCs) and tumor-associated fibroblasts (TAFs) [37] and circulating tumor cells (CTCs) [38], respectively. Exosomes secreted by CAR T-cells have been shown to elicit strong anti-tumor effects against breast cancer cells expressing EGFR and HER2 as well as mesothelin (MSLN) in vitro and in vivo [39, 40]. In vitro analysis demonstrated the presence of CAR and CD63 and MHC I proteins and CD3, CXCR4 and CD57, with undetectable amounts of CD45 RA and PD-1. More notably, the CAR-containing exosomes carried cytolytic enzymes like perforin and granzyme B and displayed substantial cytotoxic action against cancer cells unaffected by immunological checkpoints like PD-1 [41]. Another promising aspect is that intraperitoneal injection of the CAR-exosomes did not lead to cytokine release syndrome (CRS) in mice. Since exosomes can be isolated and stored as off-the-shelf products, CAR exosomes represent a promising alternative to CAR T-cells for cancer immunotherapy.

### Nanotechnology for cancer vaccines

Cancer vaccines refer to vaccines that either i) prevent the viral infections that lead to the development of certain cancer (e.g., cervical cancer by human papillomavirus (HPV)) or ii) prevent or treat the cancers in high-risked individuals, known as a prophylactic and therapeutic vaccine, respectively. Currently, the two major challenges in cancer vaccine development are the high variability of the tumor-associated antigens (TAA) in different tumors, and the immunosuppressive TME [42]. Consequently, the careful selection of TAA as a cancer vaccine will dictate such vaccine's efficacy and safety. Tumor lysates encompass the full array of TAA and can elicit potent anti-tumor immunity [43]. Adjuvant and combination immunotherapies with peptide or nucleic acid-based vaccines are being investigated as potential ways to bolster stronger and longer-lasting immune responses against cancer cells [44, 45].

#### Nanotechnology in peptide-based vaccines

**Fig. 3:**
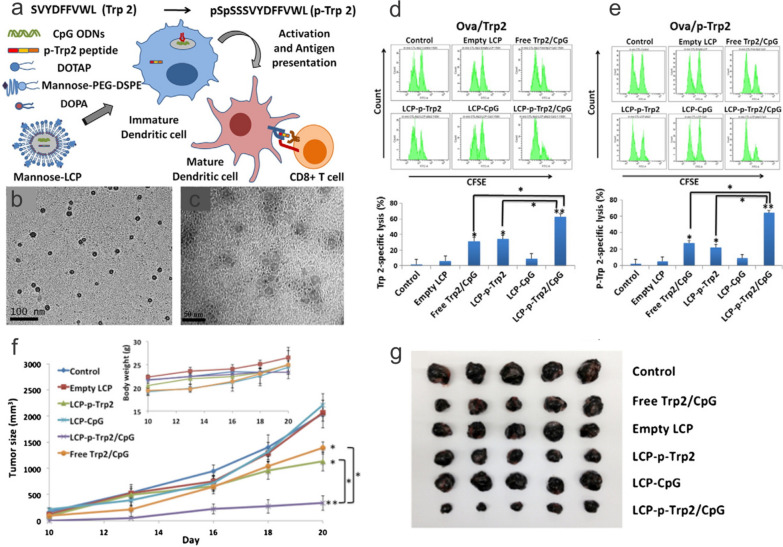
Lipid calcium phosphate nanoparticle (LCP NP)-mediated co-delivery of Trp2 peptide and CpG in B16F10 subcutaneous tumor. **a** Synthesis LCP NPs that encapsulate phosphorylated Trp2 (p-Trp2**)** and CpG for the delivery into DCs via the mannose receptor. TEM image of **b** hydrophobic LCP cores and **c** mannose-functionalized, aqueous LCP NPs. In vivo CTL response assay examining mice immunized with control peptide/CpG or mannose-LCP NPs containing both Trp2/CpG against splenocytes pulsed with **d** Trp2 or **e** p-Trp2. **f**, **g** Reduction of B16F10 tumor sizes in mice immunized with LCP NPs containing both Trp2 and CpG. Reproduced with permission from ref [47]

Successful eradication of tumors requires the generation of MHC I-restricted cytotoxic T lymphocytes (CTLs). This is achieved via delivering TAA as a peptide or gene in combination with the robust stimulation of DCs, which can further activate TAA-specific T cells. Tyrosinase-related protein 2 (Trp2) has been identified as a TAA of melanoma, and the delivery of the epitope peptide (SVYDFFVWL) has been adapted in different nanoplatforms [46, 47]. Tsai et al. developed a simple polyplex formulation by mixing arginine-modified Trp2 with CpG at various ratios. This approach allowed for the interrogation of the role of individual vaccine components in the immune system in a “carrier-free” manner. While a ratio of 5:1 Trp2R_9_ to CpG led to the highest antigen loading and greatest uptake in DCs, the expression of activation markers including CD40, CD80, and CD86 were less than the DCs treated with free CpG. This could be explained by the R9's stronger binding to the CpG, resulting in less release and stimulation of Toll-like receotir (TLR) 9. Nonetheless, complexing with Trp2R_x_ led to greater T cell proliferation and IFN-γ release as well as a reduced tumor burden. Alternatively, both the Trp2 peptide and CpG could be co-encapsulated into LCP NPs for efficient cytosolic delivery into DCs (Fig. 3). Interestingly, one of the lipids used in the study, dioleoyl-3-trimethylammonium propane (DOTAP), also possesses immuno-stimulating properties such as upregulating the production of cytokines and enhancing the cross-presentation of antigen by DCs in addition to serving as a carrier material. These capabilities are mediated through the induction of reactive oxygen species and activation of TLR4 intracellularly. Cationic nanoparticles are also efficiently taken up by DCs when compared with other cell types. Because DCs are critical antigen-presenting cells (APCs) primarily responsible for starting T cell immune responses, cationic nanoparticles' rapid absorption, together with their immunostimulatory properties, could potentially increase the immunogenicity of cancer vaccines. The composition of cationic nanoparticles carrying cancer vaccines can be greatly modified and varied with different types of antigens, excipients, adjuvants, and material components [48]. This design flexibility makes cationic nanoparticles a promising platform for vaccine delivery and offers endless possibilities for further optimization.

Polymeric nanoparticles composed of poly(D,L-lactide-co-glyoclide) (PLGA) are also promising TAA delivery platforms. PLGA is a biocompatible polymer and has a well-established safety profile. Nanoparticles fabricated with PLGA are small enough to be administered via conventional vaccine routes (subcutaneous, intramuscular). This quality is important when discussing the delivery of TLR7/8 agonists, such as peptide/protein-based TAAs, which are typically limited by their poor retention at the injection site. TLR7/8 agonists are cytokine inducers that can be used as cancer adjuvants to activate DCs and incite a robust T cell response. A platform, such as PLGA nanoparticles, that could improve their availability and exposure to DCs may provide great immunogenic improvements. Studies suggest that using nanoparticles to encapsulate these peptide-based TAAs and their adjuvants provides protection from degradation and enhanced/targeted delivery to DCs. This can ultimately strengthen T cell response reactions. Because TLR7 and 8 are located on the luminal side of endo/lysosomes, peptide TAAs must be effectively delivered through the cellular membrane and internalized into these endo/lysosomes to incite an immune response. PLGA nanoparticles are a favorable delivery platform for these TLR agonists since they efficiently enter these endosome/lysosomes after being endocytosed into the cell. Another reason why PLGA nanoparticles are efficient as in vivo vaccine delivery vehicles is because they prevent the rapid clearance of antigens from the injection site. This is accomplished by protecting their payloads from biodegradation and efficiently directing themselves to lymph nodes rather than to systemic circulation where they are rapidly cleared [49]. These properties demonstrate how PLGA nanoparticles encapsulating TLR7/8 agonists can be used to improve cancer vaccines.

#### Nanotechnology in nucleic acid-based vaccines

Although the use of these peptide-based antigens as cancer vaccines in clinical trials has demonstrated fewer side effects than conventional therapies, they have shown to provide moderate therapeutic benefits in only a small portion of patients. The risk of tumorigenesis, the threshold concentration of TAA needed for stimulation, and the presence of immunosuppressive cytokines such as IL-10 and TGF-β that can offset proper anti-tumor response preclude the wide use of tumor lysates for vaccination [50]. Genomic sequencing allows the identification of neoantigens that can overcome the aforementioned variability challenge; for example, autologous DCs can be loaded, or “pulsed” with the neo-antigen identified using high-throughput sequencing and readministered back to the patient, where they can migrate to the lymph nodes to present antigens and activate T lymphocytes [51]. Exosomes harvested from DCs have been successfully applied as cancer vaccines [52]. However, these personalized vaccines tend to be costly and time-consuming. By combining the strength of genomic sequencing technology with nanoengineering, in situ vaccination can be achieved to develop personalized DNA or mRNA cancer vaccine against specific TAA to increase both safety and efficacy. One of the most common methods of in situ vaccination is using an oncolytic virus, but systemic activation leading to CRS is a major safety concern. In situ vaccination with nonviral pDNA or mRNA vaccines delivered via nanocarriers is a safer and more cost-effective method than traditional vaccination methods. This concept has recently been demonstrated using lipidoid nanoparticles [42]. A key advantage of pDNA/mRNA vaccines is that they elicit both CTLs and helper T cells simultaneously via both MHC class I and II pathways [53, 54]. Another advantage is that multiple antigens can be encoded, allowing greater immunization [55].

The ability to rapidly design the gene construct, the relatively low cost for large-scale manufacturing, high stability and hence ease of storage, and the capacity to induce expression of target antigen for a more-extended period make DNA vaccines ideal compared to mRNA vaccines. It has been shown that plasmid DNA can persist in muscle cells for up to six months [56]. Moreover, expression of the gene endogenously will allow post-translational modifications of native protein conformation suitable for antigen presentation. While early reports demonstrated the potential of DNA as vaccines for generating tumor-specific immunity in *vivo*, the efficacy of DNA vaccine did not translate in human clinical trials. One key limitation is the low immunogenicity of DNA vaccines [57]. Specifically, the low gene transfer efficiency of DNA in APCs such as DCs is a major underlying challenge. Successful gene transfer is vital for efficiently generating MHC Class I-restricted CTLs efficiently since somatic cells like myocytes lack the MHCII or co-stimulatory molecules for T cell priming following intramuscular injection [58, 59]. The CTLs are critical in eliminating TAA-expressing cells. In addition, the DCs can further activate CD4^+^ T lymphocytes, which can assist activation of CTLs and promote the generation of CD8^+^ memory cells. To enable expression, various types of polymer and lipid platforms have been used to complex with pDNA for therapeutic vaccine applications. This includes chitosan [60], PLL [58], PEI [61, 62], PBAE [63].

Earlier works focusing on chitosan with DNA have shown the potential of inducing an immune response [64]. Furthermore, the mucoadhesive property of chitosan makes it well suited for delivery to mucosal sites such as the airway [65]. PLL is another cationic material that has been widely utilized for plasmid DNA vaccine development. Interestingly, it has been shown that PLL-coated, 40–50 nm polystyrene nanoparticles could readily transfect DCs compared to 1 µm sized particles in vitro. Moreover, both 20 nm and 1 µm particles could not generate a proper immune response, while the 50 nm particles elicited the strongest immunogenicity. The authors attributed this result to the differential uptake pathway, and noted that different materials of similar sizes, such as gold and silica, could produce similar effects [58]. Many works have incorporated mannose for targeted delivery to increase the uptake and hence the total efficacy of the DNA vaccine since DCs also express MRC1 like macrophages [66]. In a separate study, PEI was used to condense OVA-encoding pDNA for delivery into DCs [62]. Specifically, the role of PEI as a cancer vaccine adjuvant was investigated. It was found that the DCs were successfully transfected and migrated to the draining lymph nodes in vivo. The animals treated with PEI/DNA had an increased CTL activity and a reduced tumor growth. The vaccinated animals also had increased inflammation and cell death in the injection site, which could be partially attributed to PEI-mediated cytotoxicity [67]. Unlike mRNA, however, DNA must traffic to the nucleus and cross the nuclear membrane to be transcribed. Furthermore, as endosomes escape, free DNA becomes dissociated from the carrier material and vulnerable to nuclease or cytosolic DNA sensor-mediated destruction, especially in professional APCs like DCs and macrophages [68–70]. Hence, strategies that aim at protecting the DNA cargoes after endosome escape and increasing nuclear transport of the pDNA should considerably improve transfection efficiency [71, 72].

Compared to DNA vaccines, mRNA vaccines require higher maintenance for storage and transport due to their instability from the presence of the 2’ hydroxyl. The relative ease of delivery and expression (since DNA vaccine has the additional nuclear membrane to overcome) and the transient nature have attracted attention in the last decade as a vaccine candidate over pDNA. Regardless of whether pDNA or mRNA, the delivery of these nucleic acids is often accompanied by a nanoparticle to carry this genetic payload. Although other delivery vehicles exist for mRNA vaccines, as will be discussed in a later section, many of the current nanocarriers of clinical importance are composed of lipids. In this regard, emphasis will be placed on the design of the lipid nanoparticles as well as the selection of their compositions. It is important to note that lipid nanoparticles differ from liposomes in that liposomes have at least one lipid bilayer with an aqueous core, whereas lipid nanoparticles have a presence within the core as well. When observing the composition of clinically relevant lipid nanoparticles, there are often 4 main components that include (i) neutral phospholipids, (ii) ionizable cationic lipids, (iii) cholesterol, and iv) polyethylene-glycol (PEG)-lipids. The neutral phospholipids and cholesterol aid in the overall structure and stability of the lipid nanoparticle, while the ionizable cationic lipids and PEG-lipids provide more functionality and improve storage conditions. Cationic ionizable lipids are composed of three parts, (i) an ionizable head group, (ii) a linker region, and (iii) lipid tails. The ionizable head group and linker region both facilitate endosomal escape and contribute to the overall pKa, which has been shown to elicit an optimal adaptive immune response when tailored to 6.6–6.9 for mRNA vaccines [73]. The lipid tails influence the geometry, and thus endosomal escape capabilities, in addition to the toxicity and storage conditions of the lipid nanoparticle [74]. For example, by incorporating ester linkages into the hydrocarbon tail, the lipid nanoparticle can degrade quicker because of the cleavage caused by the metabolic activity of esterases present in the cell, but placing these esters too close to the head group could influence the system’s overall pKa [75]. Moreover, because ionizable lipids are cationic, they can interact with anionic mRNA during particle formation to ensure that they are encapsulated in the lipid nanoparticle. PEG-lipids are often used in small molar percentages in the overall formulation but provide a steric hindrance to prevent lipid nanoparticle aggregation and contribute to the nanoparticle's overall size [76].

### Nanotechnology in adoptive cell transfer for cancer immunotherapy

#### Nanoparticles in immune cell-assisted delivery

**Fig. 4:**
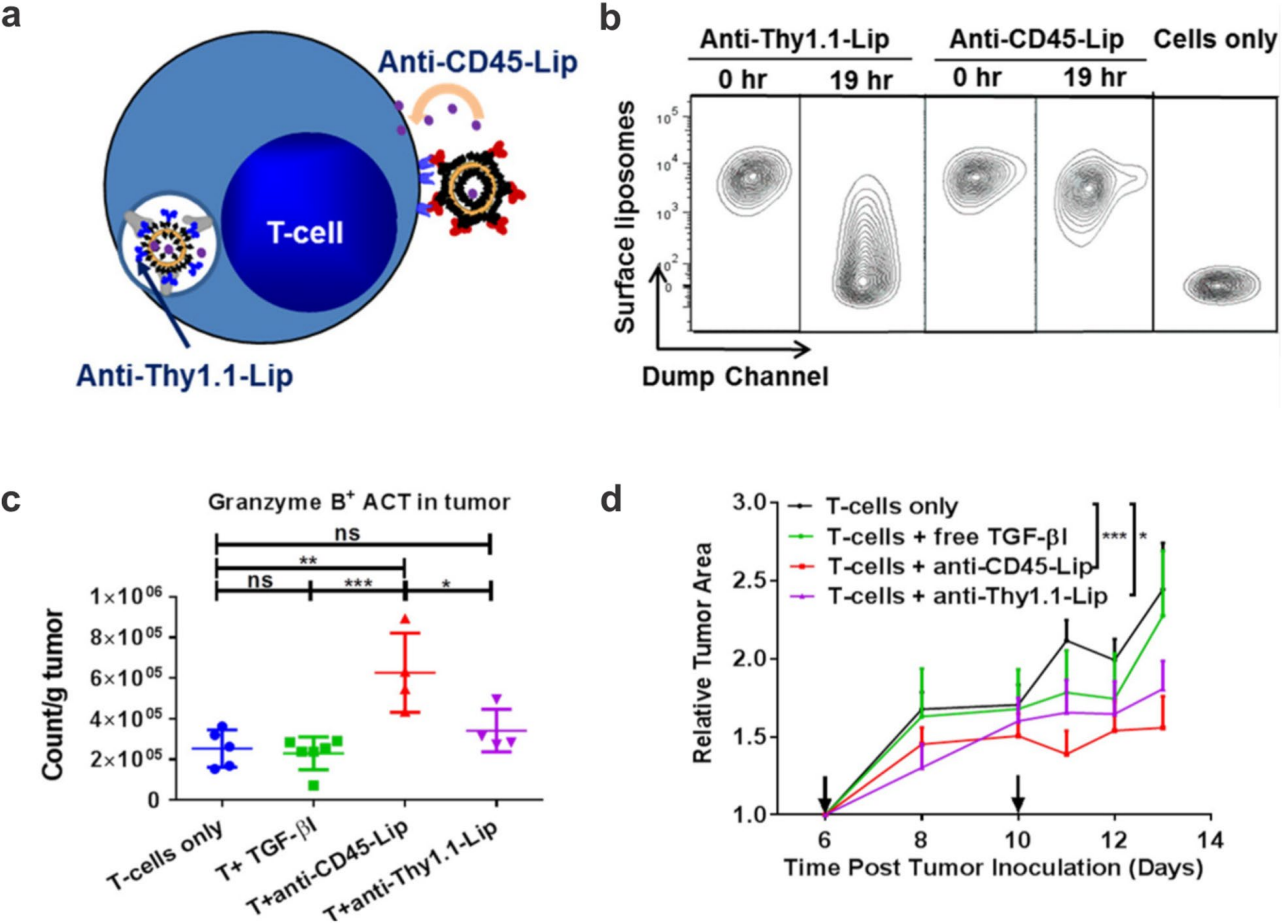
Surface engineering of T cells with nanoparticles. **a** Schematic diagram depicting the liposomes anchoring to either CD45 or Thy1.1 receptor on T cells. **b** Flow cytometry showed that remaining liposomes on cell surface-bound to CD45, a non-internalizing receptor, were significantly greater than those bound to Thy1.1, an internalizing receptor. **c** T cells loaded with anti-CD45 liposomes containing TGF-β inhibitors led to greater infiltration in tumors. **d** CD45 bound liposomes decreased the tumor size of B16F10 tumors compared to either free or Thy1.1 bound liposomes. Reproduced with permission from ref [81]

While therapeutic nanoparticles functionalized with PEG can enhance circulation half-life and hence overall bioavailability, the current solid tumor delivery paradigm is ineffective, with less than 1% of the dosage reaching the target [77]. One alternative approach is to functionalize drugs or adjuvants encapsulated in nanocarriers onto immune cells, such as T cells and NK cells delivery in vivo. These adjuvant nanoparticles as “cellular backpacks” have been studied extensively in T cells to enhance T cell functions [78]. Key advantages of backpacking adjuvants include (i) limiting systemic toxicity from high dosing of adjuvants and (ii) allowing small molecule drugs to be administered (that cannot be genetically expressed). In an earlier study, multilamellar liposomes (~ 300 nm) with lipids containing maleimide headgroups were developed [79]. The maleimide functional groups can readily conjugate with the free thiols on the surface of T cells and HSCs and remain for days even after stimulation in vitro; on the other hand, the maleimide-liposomes were readily internalized by immature DCs. These surface anchoring nanoparticles were nontoxic and could achieve week-long sustained release of the therapeutic payloads. Importantly, attaching these nanoparticles to the cells at ~ 100 particles per cell did not interfere with vital cellular functions in cytotoxic T cells, including proliferation, cytotoxicity, diapedesis, and tumor homing ability in vivo. To investigate the effects of adjuvant-loaded nanoparticles on CD8^+ ^T cells, IL-15Sa and IL-21 were co-encapsulated into multilamellar lipid nanoparticles and anchored to the T cells. These backpacked T cells showed long persistence in mice with melanoma lung and bone marrow tumors. Impressively, the surface anchored nanoparticles provided 11-fold enhancement in the immunostimulatory effects to the CD8^+^ T cells compared to that by co-administered, non-attached nanoparticles. Using a similar strategy, Stephan et al. encapsulated NSC-87877, a small molecule inhibitor of SHP1 and SHP2, into stable liposomes containing hydrogenated soybean phosphatidylcholine and cholesterol and studied the spatial distribution of the nanoparticles on the effector T cells [80]. Interestingly, the nanoparticles were discovered to be localized in the uropod during migration but concentrated to the immunological synapse upon cell target recognition. This phenomenon did not interfere with the killing of target tumor cells. The encapsulated drug was found to be released slowly over 6 days, during which the tumor-specific T-cells could expand. Infusion of the nanoparticle-modified T-cells showed significant tumor infiltration and extended survival in a prostate cancer mouse model. The authors used mass spectrometry to identify CD45 as the principal membrane anchor for the nanoparticles linked to the T cell surface, as well as adhesion proteins (LFA-1, CD2, CD97), CD98, the transferrin receptor, and the MHC-1 complex, to mention a few. Zheng et al. prepared liposomes containing the hydrophobic TGF-β inhibitor (TGF-βI) SB525334 using the ethanol injection method and anchored the particles to the pmel-1 CD8^+^ T cells via CD45 (non-internalizing receptor) or CD90 (Thy1.1, internalizing receptor) (Fig. 4) [81]. It was found that ACT T cells pre-loaded with CD45-targeting TGF-βI liposomes ex vivo led to superior tumor infiltration of ACT T cells than those backpacked with CD90-targeting liposomes and reduction of tumor size in a B16F10 melanoma murine model. This CD45-targeted loading strategy was later adopted for the loading of stimulus-responsive nanogels containing interleukin IL-15 super-agonist (IL-15Sa) [78] and IL-2 [82] onto T cells for better drug encapsulation and regulation of release. Table 1 summarizes the surface functionalization of nanoscale dimension cargoes onto various types of immune cells.

**Table 1:** Surface engineering of immune cells with nanomaterials for delivery

Cell Source	Cargo	Size	Functionalization Strategy	Application	References
T cell	Multilamellar liposomes	~ 300 nm	Maleimide-cell surface thiol	B16F10 melanoma lung and bone marrow tumors	[79]
	Lipid-coated PLGA	~ 230 nm	Maleimide-cell surface thiol	B16F10 melanoma lung and bone marrow tumors	[79]
	Liposomes	~ 200 nm	Maleimide-cell surface thiol	Prostate tumor	[80]
	Lipid nanocapsules	~ 340 nm	Maleimide-cell surface thiol	Lymphoma cells	[83]
	Liposomes	~ 83 nm	CD45 antibody conjugation	B16F10 melanoma	[81]
	Cytokine nanogels	~ 100 nm	Covalent conjugation via crosslinker/ electrostatic interaction	B16F10 melanoma	[82]
	Lipid nanocapsules	~ 240 nm*	Maleimide-cell surface thiol	Functional modification of CTLs	[84]
CAR T-cell	Cytokine nanogels	~ 80–130 nm	CD45 antibody conjugation/ electrostatic interaction	B16F10 melanoma	[78]
	Multilamellar liposomes	~ 160 nm*	Maleimid-cell surface thiol	SKOV 3 ovarian cancer and leukemia	[85]
NK cell	Liposomes	~ 138 nm	NK1.1 antibody conjugation	SW620 colon cancer cells	[86]
	Graphene oxide-PEG nanoclusters	~ 50–300 nm	CD16 antibody conjugation	Activation of NK cells	[87]
CAR NK- cell	Liposomes	~ 220 nm*	Maleimide-cell surface thiol	SKOV 3 Ovarian cancer	[88]
Leukocyte	Liposomes	~ 118 nm	Binding between E-selectin receptor and apoptosis inducing ligand TRAIL	Circulating colon cancer cells (COLO 205)	[89]
B cell	Multilamellar Liposomes	~ 300 nm	Maleimide-cell surface thiol	B16F10 melanoma lung and bone marrow tumors	[79]
HSC	Multilamellar Liposomes	~ 300 nm	Maleimide-cell surface thiol	B16F10 melanoma lung and bone marrow tumors	[79]

*Size values taken from previously reported literature sources [90, 91]

#### Nanoparticles in car immune cell manufacturing

**Fig. 5:**
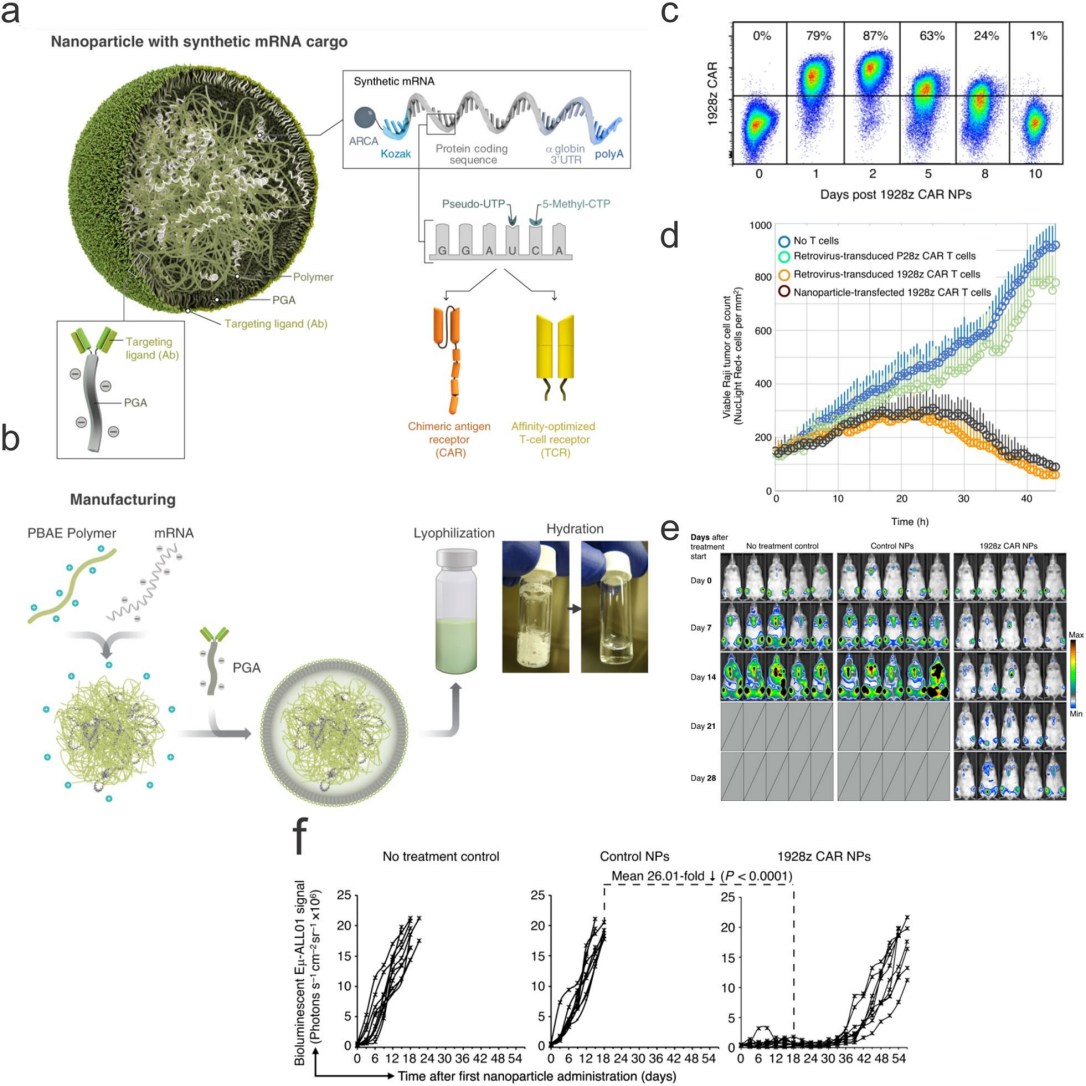
Polymeric nanocarrier for the delivery of CAR IVT mRNA into T cells. **a** Schematic illustration of the PBAE nanocarrier encapsulating the IRF5 and IKK IVT mRNA. The polymeric nanoparticles created from self-assembly could be readily lyophilized and redispersed, increasing flexibility for storage and transport. **c** Transfection efficiencies of the nanocarriers into T cells. **d** The nanoparticle-reprogrammed T cells showed comparable lysis activity to lentivirus-transduced T cells against Raji lymphoma cells. **e** Only the transfected T cells were able to alleviate tumor burden and **f** extend average survival time in C57BL/6 mice. Reproduced with permission from ref [99]

One important application of nanotechnology for the engineering of immune cells (T cells, NK cells, and macrophages) with CAR is the nonviral delivery of the CAR constructs, either in the form of DNA or RNA, into these cells. While viral vectors demonstrate superior gene transfer efficiency, with approximately 70% of clinical trials using viral vectors such as lentiviruses and adenoviruses [92], limitations associated with viral vectors such as immunogenicity, tumorigenicity, limited packaging capacity, and difficulty to scale continue to plague viral vector application for successful commercialization [93–95]. On the other hand, nonviral vectors offer advantages such as better safety profiles, reduced immunogenicity, greater loading capacity, and ease of scaling [96, 97]. In particular, nonviral vectors allow for T cells' in-situ programming, which significantly reduces the time and cost of preparation for ACT T cells. This is demonstrated by the Stephan group, who designed an anti-CD19 194-1BBz CAR encoded in the piggyBac transposon that was encapsulated in a PBAE nanocarrier modified with a peptide containing the microtubule-binding and nuclear localization sequence [98]. The cationic particle core was further coated with PGA-functionalized with anti-CD3e for targeting T cells in vivo. The size of the polymeric nanoparticles was approximately 150 nm in size and -8 mV in zeta potential. While the in vitro and in vivo transfection efficiencies were low (~ 3% and ~ 1.5%, respectively), the engineered T cells showed enhanced tumor lysis and cytokine production compared to those transduced with lentiviral vectors in vivo. The same group later demonstrated the delivery of in IVT mRNA encoding anti-CD19 1928z CAR using the same PBAE nanocarrier formulation [99] (Fig. 5). The anti-CD8 antibody functionalized nanocarriers were readily bound to T cells and demonstrated superior transfection efficiency (~ 75%) in vitro compared to the plasmid-encapsulating nanocarriers. The reprogrammed CAR T-cells could readily recognize the CD19 + Raji cells, completely eradicate the tumors, or lead to significant tumor regression. The potential of this technology was further demonstrated in mice bearing solid tumors, in which nanoparticle-reprogrammed CART-cells expressing antiROR1 could efficiently eliminate LNCaP C4-2 prostate tumor cells and extend the survival of mice by 42 days. However, compared to stably transfected or transduced T cells, IVT mRNA transfected CAR T-cells showed transient expression of 1928z CAR for about 8 days. Nonetheless, repeated dosing of the mRNA nanoparticles could reach the same levels of gene transfer (~ 10%) into the host T cells, demonstrating that this approach could still lead to long-term expression of CARs on T cells. Regardless of whether the cargo is DNA or RNA, one major advantage of these polymeric nanocarriers is that they can be lyophilized and reconstituted, offering the potential as an off-the-shelf product for storage and transport [100].

Like in situ vaccination, the critical factor that limits the use of this approach is the gene transfer efficiency, especially for DNA, in host T cells. Primary T cells are refractory to transfection. Tailoring the structural properties of polymeric or lipid-based materials could lead to better gene transfer into T cells and therapeutic efficacy overall. For example, Olden et al. investigated a library of sunflower and comb-shaped poly(2-dimethylaminoethyl methacrylate) (pDMAEMA) for T cell transfection. They found that one of the candidates, l-pHEMA25-g-(pDMAEMA16)25 (CP-25–16), could achieve a reporter gene transfection efficiency of ~ 25% with mRNA and ~ 18% with DNA in human primary CD4^+^ and CD8^+^ T cells while maintaining high (> 90%) viability in vitro [101]. In another study, Richter et al. developed a block copolymer micelle incorporating lipoic acid into poly{[2-(dimethylamino)ethyl methacrylate]_101_-*b*-[*n*-(butyl methacrylate)_124_-*co*-(lipoic acid methacrylate)_22_]} (p(DMAEMA_101_-*b*-[*n*BMA_124_-*co*-LAMA_22_])) and showed ~ 29% transfection efficiency in K-562 cells while cells transfected with pDMAEMA showed negligible expression of the reporter [102]. Ayyadevara et al. had found that the mere addition of Ca^2+^ could increase the transfection of polyplex into Jurkat cells compared to polyplex-only control, while other cations such as Na^+^ or Mg^2+^ did not lead to such effect [103]. The increase in transfection was partially attributed to the increased association of polyplex with the cell membrane.

In addition to polymeric-based nanocarriers, lipid nanocarriers have also been investigated to deliver CAR genes into T cells. Lipids represent one of the most widely used materials as vectors for nonviral gene transfer. This is because they are generally considered safe, biocompatible with low immunogenicity, and easy to use. In contrast to polymeric nanoparticles, lipid nanoparticles are more clinically developed for the delivery of RNA [104]. Many of the cationic lipids developed for transfection have positively charged head groups and hydrophobic tails connected by a linker, which is either an ester, ether or amide functional group that determines the overall flexibility and biodegradability of the cationic lipid [105]. Some examples of the widely used cationic lipids for transfection include (N-[1-(2,3-dioleyloxy)propyl]- N,N,N-trimethylammonium chloride) (DOTMA), (2,3- dioleyloxy-N-[2(sperminecarboxamido)ethyl]-N,N-dimethyl1-propanaminium trifluoroacetate) (DOSPA), and (N-[1-(2,3- dioleoyloxy)propyl]-N,N,N-trimethylammonium methyl sulfate) (DOTAP) [97]. They can form complexes spontaneously in the aqueous environment with the negatively charged nucleic acids through the positively charged head groups. However, since cationic lipids tend to be cytotoxic, researchers have adopted ionizable lipids (lipids that are neutral at physiological pH but become positively charged at lower pH) for clinically relevant lipid nanoparticle formulations [106–108]. Billingsley et al. synthesized and evaluated a library of ionizable lipids, and found that the top candidate, C14-4, when formulated with 1,2-dioleoyl-sn-glycero-3-phosphoethanolamine (DOPE), cholesterol, and C14-PEG, was able to induce the greatest luciferase expression in Jurkat cells and primary CD4^+^ and CD8^+^ T cells without increased cytotoxicity compared to Lipofectamine [109]. The authors then assessed the delivery of IVT CD19 CAR mRNA into primary T cells using the same formulation and compared it with electroporation, the most commonly used method for the nonviral delivery of CAR in vitro and ex vivo. The C14-4 formulation exhibited comparable CAR expression to electroporation when measured by flow cytometry, but much less cytotoxicity. Importantly, the CAR T-cells generated by the ionizable lipid nanocarriers demonstrated cancer-killing activity on par with those transfected by electroporation and transduced by lentiviral vectors in a co-culture assay with Nalm-6 acute lymphoblastic leukemia cells. The same group further screened and identified additional candidates A16 and B10 as promising ionizable lipid formulation for functional delivery of mRNA into primary T cells with low cytotoxicity [110].

Besides T cells, NK cells also have received increased attention as the source for CAR engineering. One key advantage is that transplanted NK cells do not give rise to Graft Versus Host Disease (GvHD) due to the lack of T cell receptors (TCRs). Therefore, attempts to generate off-the-shelf, allogenic CAR NK cells have received a lot of research focus. In one instance, Kim et al. has developed a core–shell iron oxide nanoparticle platform for the transfection of CAR NK cells [111]. The core is made of zinc-doped iron oxide layered by caffeic acid and polydopamine, followed by an outer layer of PEI. The nanoparticle was able to deliver both EGFP and CAR encoding EGFR into NK-92MI cells. Importantly, the transfected NK-92MI cells could elicit cytotoxicity against MDA-MB-231 cells in vitro and in vivo. In addition to acting as a nanocarrier for gene delivery, the iron oxide nanoparticle also enabled MR imaging in vivo as an excellent T2 contrast agent. This allowed tracking of the CAR NK cells once administered. In contrast to conventional CAR design, which contains the scFv against the target antigen, Wang et al. engineered CAR NK cells with a piggyBac construct encoding NKG2D as the extracellular domain, and DAP10 and CD3ζ as the co-signaling domain via the biodegradable PBAE nanocarrier [112]. Compared to the control NK-92 cells, the transfected NK-92 cells exhibited greater degranulation activity and IFN-γ production, as well as cytolysis against solid tumor cell lines GBM43, GBM10, A549 and PC3. The cytolysis activity of the NKG2D.CAR-NK-92 cells were further enhanced by CD73 blockade both in vitro and in vivo.

While T cells and NK cells can exert powerful anti-tumor activities, they are susceptible to the immunosuppressive TME, rendering them relatively ineffective for solid tumors. On the other hand, Macrophages are resilient and abundantly present in many solid tumors due to the active recruitment of bone marrow-derived monocytes to the TME and subsequent differentiation and polarization to the pro-tumoral (M2) phenotype. Taking advantage of this phenomenon, Klichinsky et al. developed CAR macrophages (CAR-Ms) using an adenoviral vector (Ad5f35) that could elicit a high degree of gene expression, since macrophages are very difficult to transfect with high efficiency [113]. In multiple cancer models, CAR-Ms displayed direct anti-tumor effects in vitro and in vivo; additionally, these altered macrophages were engaged in antigen cross-presentation and activating T cells in vivo, which is crucial for fostering active anti-tumor immunity. The switch towards a pro-inflammatory (M1) state upon antigen recognition and involvement in antigen presentation were similarly found in CAR-Ms generated from iPSCs via GSEA analysis [114, 115]. The TAMs could be redirected to a CAR-expressing M1 state using a nonviral, piggyBac vector encoding an anti-ALK CAR and the IFNγ gene. The CAR-Ms reprogrammed in situ mirrored the ACT CAR-Ms reported by Klinchinsky et al. They exerted anti-tumor activity via CAR-mediated phagocytosis, presented antigens, and activated CD8^+^ T cells. However, as with the other nanoparticle-based in situ programming approaches, the gene transfer efficiency was significantly lower than that of viral vectors, indicating that achieving high gene transfer efficiency in these immune cells remains a critical bottleneck that, if overcome, could lead to significantly improved therapeutic outcomes. Nevertheless, CAR-Ms represent a promising avenue for tackling solid tumors, and that compared to CAR T-cell therapy, which can lead to CRS, the pro-inflammatory cytokines generated by CAR-Ms are more confined to the TME, further reducing the risk of CAR-based therapies.

#### Nanotechnology for modulating car T-cell activity

Nanotechnology also offers ways to manipulate CAR T-cell activity besides acting as delivery vehicles for gene transfer. One of the challenges associated with CAR T-cell therapy is the adverse effect associated with CRS because of the lack of control of activated T cells activity. A conventional approach to modulate such activity is by engineering ON and OFF switches in CAR T-cells [116–118]. Alternatively, nanoparticles with intrinsic material properties can be tailored to manipulate the activation or deactivation of the CAR constructs. By harnessing these properties, including the material’s propensity to generate or respond to light, heat, or magnetic field, the state of the CAR T-cells can be tuned to reduce off-target effects and CRS. Nguyen et al. developed a light-switchable CAR (LiCAR) that could only be activated by a blue light when the CAR T-cells engaged the target ligand. This is accomplished by including photoresponsive domains into the split CAR constructs; upon illumination and binding to a substrate, the split CAR constructs dimerize and activate intracellular signaling pathways [119]. The core–shell upconversion nanoplates (UCNPs) acted as light transducers for LiCAR, in which the Yb^3+^ served as the sensitizer and Tm^3+^ as the emitter for the NIR light. The dimensions of the UCNPs (~ 200 nm in diameter and ~ 85 nm in height) allowed for enhanced upconversion luminescence, which is crucial for in vivo application. To increase the efficiency of the LiCAR/UCNP system, the authors coupled streptavidin-functionalized UCNP to the LiCAR T-cells, and observed superior cancer-killing compared to tumors protected from light or treated with LiCAR T-cells without UCNP. Importantly, the LiCAR/UCNP system reduced the two major adverse effects commonly associated with conventional CAR T-cell therapy: the “on-target, off-tumor” cytotoxicity and CRS. Mice treated with LiCAR T-cells exhibited reduced B cell aplasia, a common side effect associated with anti-CD19 CAR T-cell therapy, compared to mice that received WT CAR T-cells. Furthermore, SCID-beige mice (to model CRS) treated with LiCAR T-cells showed no weight loss and reduced serum IL-6 levels when compared to mice treated with WT CAR T-cells. These results indicated that the LiCAR/UCNP system could offer a greater safety profile using the optical properties of nanomaterials for controlling cellular behavior. To further validate the utility of optical nanomaterials for CAR T-cell engineering, Miller et al. designed thermal-responsive gene switches governed by the HSPB1 core promoter that allows for CAR expression at elevated temperatures (40–42 °C) [120]. Combined with plasmonic gold nanorods injected intravenously into the tumor-bearing mice and exhibiting photothermal effects upon NIR light (650–900 nm) exposure, the thermal-activated TS-Fluc αCD19 CAR T cells could achieve targeted elimination of CD19 + Raji cells in a spatially confined manner. However, while the light is an attractive source for stimulus-responsive control, tissue penetration of light and water absorption are still major issues [121, 122], especially in larger hosts. This issue is further exacerbated in solid tumors, in which CAR T-cells are already inefficient against. Future efforts into nanomaterial-CAR T-cell platforms focusing on using stimuli that do not readily interact with body tissues, such as magnetic fields, could provide greater therapeutic efficacy. For example, anchoring magnetic nanoclusters to T cells and using MRI guidance could increase the accumulation of ACT T cells [123].

**Fig. 6:**
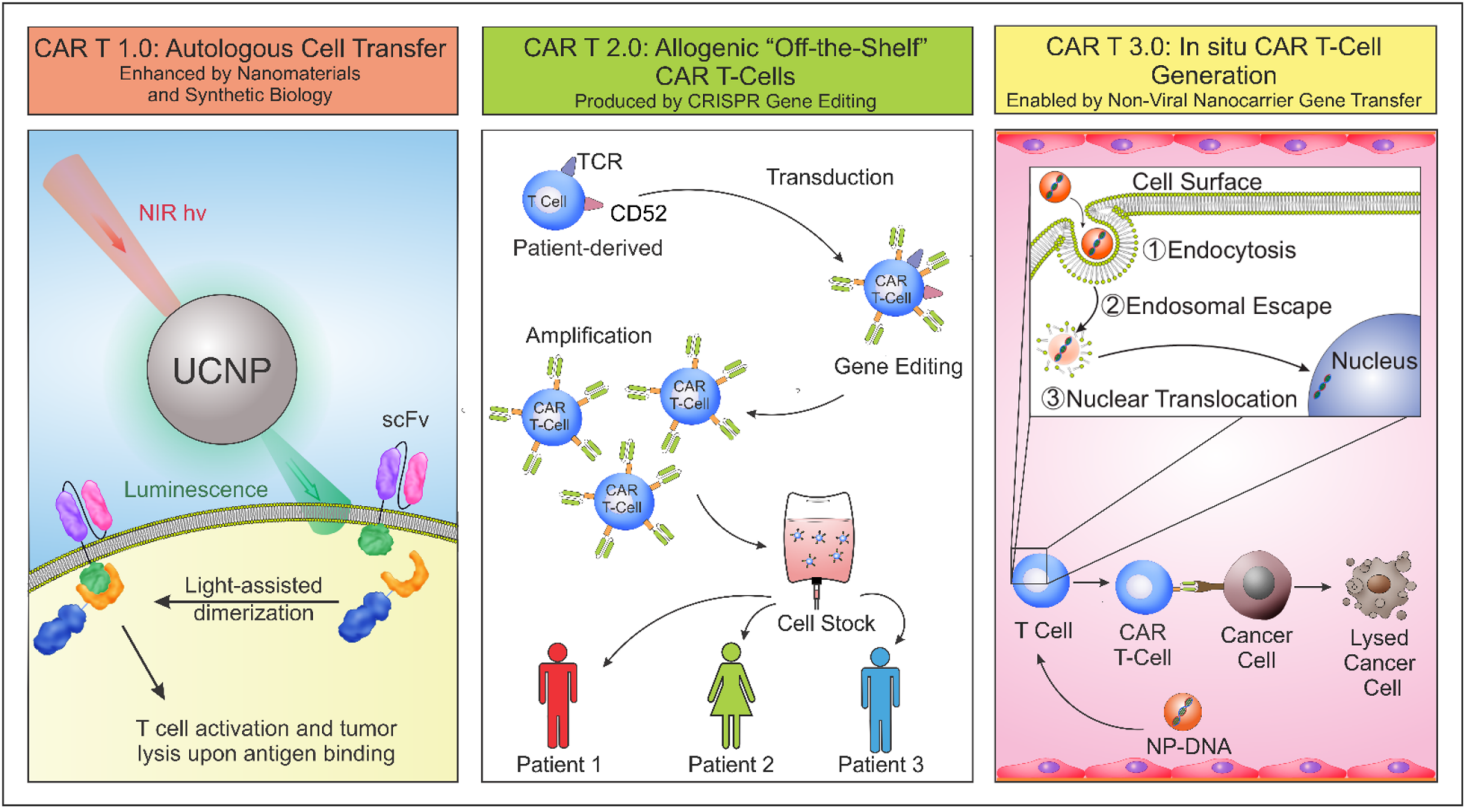
Next-generation approaches for improving CAR T-cell therapy. Advances that combine synthetic biology and optogenetics allow the design of switchable CAR T-cells with greater specificity and spatiotemporal control of activity to improve current autologous ACT (CAR 1.0). A step forward would be to engineer CAR T-cells with CRISPR multiplex gene editing to inactivate or knockout genes such as *TRAC* and *CD52* (CAR 2.0). The edited cells can then be expanded and stored as an “off-the-shelf” product. To decrease the time and costs associated with ACT, a potential direction is to adapt an in situ CAR T-cell manufacturing approach to reprogram endogenous T cells into CAR T-cells (CAR 3.0). Combined with genomic sequencing technology, this can be a personalized approach with increased efficacy and decreased overall costs

CAR-based therapies show great promise for oncology-immunotherapy. Current CAR T-cell therapy is based on autologous T cell engineering, which can be costly, and patients can experience CRS or neurotoxicity. The off-targeting effects can be minimized by including more complex designs and incorporating logic gates and nanomaterials (CAR 1.0) [119]. To further improve the therapy for wider patient access and eliminate GVHD, the CAR T-cells can be modified via CRISPR gene editing to remove the TCR alpha constant (TRAC) locus and the CD52 (CAR 2.0) [30, 124]. However, since this is a “universal product”, each patient might respond differently, hence affecting final patient outcome. To decrease the time and costs associated with ACT, a potential direction is to adapt an in situ CAR T-cell generation approach to reprogram endogenous T cells into CAR T-cells (CAR 3.0) [98]. This can be a personalized approach with increased efficacy and decreased overall costs combined with genomic sequencing technology (Fig. 6).

## Inducing acute immunity towards infectious diseases

Infectious diseases pose a foremost threat to global health and are the leading causes of death for individuals living in poverty. Despite this fact, there are limited treatment options for many of these diseases, therefore safe and efficacious vaccines only exist for a small portion of all diseases. A vaccine's ultimate purpose is to generate a high affinity and antigen specific antibody response against the immunization agent. This is usually observed by detecting an increase in IgG or IgA, which are the dominant types of antibodies produced by memory B cells following vaccination. Typically, vaccine platforms entail either live attenuated or whole inactive vaccines. Live attenuated vaccines have been available since the 1950’s and were derived from the disease-causing pathogen that has been weakened under laboratory conditions to cause either no or mild disease effects while offering the individual immunity to the pathogen. These attenuated pathogens can replicate within the host, and the stimulation of these pathogens provides enough time for memory cells to be produced if the individual is ever exposed to the pathogen. Live attenuated vaccines tend to be long lasting due to the formation of memory cells; common diseases that use this approach include tuberculosis, polio, measles, and influenza [125–127]. Conversely, inactivated whole-cell vaccines utilize pathogens that have been killed through either physical or chemical means such that they cannot cause disease. Even though this technique is regarded as safer since there are no live components, inert whole-cell vaccines may not always provoke an immune response, and the immune response that is elicited may be short-lived and require multiple doses to be successful. Typical applications include vaccines for hepatitis A, typhoid, and influenza [128–130]. As was alluded to earlier, these conventional approaches to creating vaccines have several limitations, including a complicated manufacturing process, potentially severe side effects, and severe infections. To this end, subunit vaccines, specifically those that employ nanoparticles as vaccine delivery vehicles, have been of particular interest. Much like inactivated whole-cell vaccines, subunit vaccines do not use live components of a pathogen but only the antigenic components to elicit an immune response. Several nanoparticle platforms have been developed applying the concept of subunit vaccines and have been demonstrated to induce both humoral and cellular immune responses against the pathogen they are modified with, as described in the upcoming sections.

**Fig. 7:**
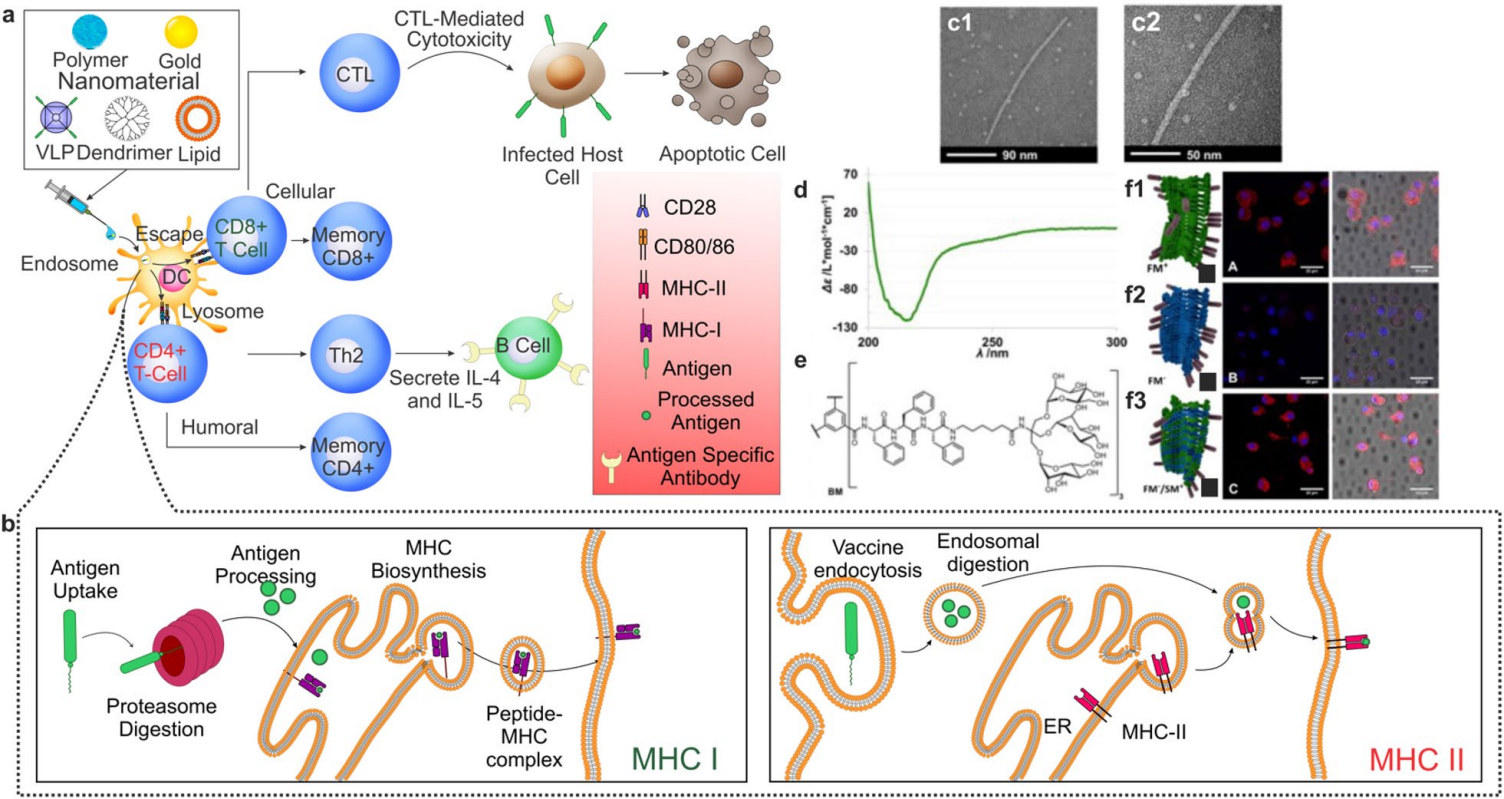
Bacterial vaccine and antigen-presenting pathways. **a** Various nanoparticles that are used to deliver antigens or antigen fragments for bacterial vaccines. Following the delivery of these antigen-bound/encapsulated platforms, the cell uptakes the vaccine via endosomal pathways. From here, the antigen can undergo processing in either the endosome, via the MHC-II pathway, or be processed by proteases in the cytoplasm before the presentation by the MHC-I complex. Antigen presentation via MHC-II results in humoral immunity where T_h_2 cells activate B cells to produce antibodies against the bacteria. Conversely, MHC-I presentation results in the differentiation of T cells into CTLs that can initiate cell death by cellular immunity. **b** Pathways for MHC-I and MHC-II antigen processing. In MHC-I presentation, the antigen can escape the endosome and is processed by proteases. The processed antigen is transported to the endoplasmic reticulum (ER), where MHC biosynthesis occurs, complexes with the MHC-I ligand, and is transported to the cell membrane for presentation to T cells. During MHC-II pathways, the antigen is digested in the endosome and fuses with MHC-II intracellularly to form the peptide MHC-II complex prior to being shuttled to the cell surface for presentation. **c** TEM characterization of negatively stained self-assembly polymer to demonstrate monodispersity and size range of mannose-decorated multicomponent supramolecular polymers targeted towards APCs. **d** Circular dichroism spectra of dendritic glycopeptide peptide. **e** Chemical structure of amphiphilic glycopeptide used as a monomer for self-assembly. **f** Cellular uptake of self-assembled polymers with and without mannosylated monomers (**f1** and **f2**) and mannosylated monomers without a fluorescent label (**f3**). Reproduced with permission from ref [135]

Despite the development of a plethora of antibiotics over the years, the treatment of bacterial infections is still plagued by several challenges, specifically owing to an increase in antibiotic-resistant bacteria strains. To this end, a wide range of nanoparticle platforms, encompassing dendrimers, liposomes, polymeric, protein, and inorganic nanoparticles, have been implemented to enhance the therapeutic effectiveness of antibiotics and their function as vaccine adjuvants. For the latter, nanoparticles are targeted towards APCs, including DCs, macrophages, and B cells to uptake extracellular proteins, process them, and present these peptides to CD4^+ ^T cells to elicit long-term humoral immune responses against the antigen in the form of antigen-specific antibodies (Fig. 7a, b). Of particular interest in the field of nanoparticle vaccines are liposomes. Liposomes are a promising delivery vehicle as their compositions can make them nontoxic, nonimmunogenic, and biodegradable. Moreover, liposomes are very modular as their size, lipid composition, charge, and loaded antigen cargo can be easily modified [131]. As such, antigens can easily be encapsulated in the hydrophilic core and protected from enzymatic degradation by a lipid bilayer, which functions to increase the bioavailability of the antigen by allowing for facile transport through the cell membrane [132].

### Bacterial infections and subunit vaccines

Several studies have highlighted the usage of liposomes as a delivery vehicle for subunit vaccination. For example, a recent study encapsulated utilized a liposome-based approach to encapsulate two tuberculosis (TB) antigens (Ag85B and ESAT-6) as a novel subunit vaccine and delivered this system into C57BL/6 mice [133]. The liposome comprised phosphatidylserine encapsulating the two TB antigens and was delivered first subcutaneously and then intranasally. It was observed that mice exposed to the liposome containing the Ag85B antigen produced large amounts of IFN-γ, which is essential for TB resistance. Following stimulation, splenocytes were discovered to overexpress IL-17A, which has been hypothesized to produce CD4^+^ cells that occupy the lung post-infection and produce chemokines that recruit IFN-γ secreting CD4^+ ^T cells to help control the infection [134].

Aside from liposomes, another promising approach to synthesizing nanoparticle vaccines is through the usage of polymers and dendrimers. Much like liposomes, polymer platforms are highly modular and versatile. By carefully tuning the monomeric units, it is possible to create biodegradable, biocompatible, and nontoxic polymeric particles as possible vaccine vehicle candidates. This idea was highlighted in a study that utilized mannose-decorated supramolecular polymers to facilitate the uptake of the platform by APCs [135]. This study used a combination of benzene-tricarboxamides and triazine-branched nonaphenylalanines to guide supramolecular polymerization, which was carried out in conjunction with mannose functional monomers (Fig. 7c–f). Although this system was not used to encapsulate an antigen, the platform could be internalized by macrophages and demonstrated the potential to be further developed in future applications. A similar system was designed in 2016 using a combination of PLGA and PEI to encapsulate a model antigen and impart a positive charge to facilitate cellular uptake and endosomal escape [136]. This platform encapsulated OVA and could induce cross-presentation of the model antigen on MHC-I molecules for a strong and antigen-specific response in CD8^+^ cells.

While less commonly used in bacterial infections, virus-like particle (VLP) vaccine platforms have also been reported in developing vaccines against anthrax infections. The VLP that will be discussed is derived from the bacteriophage *Escherichia* virus T4 (T4) and was genetically fused in their Hoc or Soc region to the antigen of interest. Anthrax infections are caused by *Bacillus anthracis*, which is a gram-positive bacterium, that can usually be treated by antibiotics but is fatal if inhaled. Recently, a *B. anthracis* vaccine was created by fusing the anthrax protective antigen (PA) to the T4 Soc capsid proteins [137]. Several animal models, including mice, rats, and rabbits, were subjected to the anthrax lethal toxin at 100% lethal dosage, and all the treated animals could survive. This was attributed to the vaccine's ability to elicit both humoral and cellular immunity in the treated animals that were notably absent in the nontreated controls, which died two days following injection.

Inorganic nanoparticles, specifically gold nanoparticles (AuNPs), have been utilized as another approach to create vaccines against bacterial infections owing to their ability to be easily functionalized and to act as an adjuvant. This is especially true with gold, which has piqued interest due to its inherent stability, low toxicity, capacity to activate macrophages, and ability to cause immune responses in lymphocytes that create antibodies against certain antigens [138–140]. In recent years, a number of studies have been conducted to develop subunit vaccines against bacterial infections using gold nanoparticles as the primary building block. One example utilizes a subunit of the flagellin of *Pseudomonas aeruginosa* as a vaccine candidate due to the presence of thiol-containing cysteine residues near the N-terminus that can form Au–S bonds [141]. This flagellum is known to interact with TLR5 to elicit an immune response. Additionally, *P. aeruginosa* is a gram-negative bacterium that causes infections in immunocompromised patients and is responsible for 10% of hospital-acquired infections. Moreover, this bacterium has exhibited multidrug resistance, making antibiotic treatment difficult or unfeasible. By conjugating the flagellum to the AuNP, the authors could create a highly immunogenic system, which would be favorable to be internalized by DCs and macrophages due to the presence of the AuNP, which resulted in the formation of antibodies that would recognize the whole bacteria. Similar studies were performed for other bacterial targets, including tetanus toxin and *Listeria monocytogenes*, where a botanical adjuvant was utilized in conjunction with the  AuNP to increase the systemic response of IgG and IgA in one study. In a separate study  an Au glyconanoparticle with a listeriolysin O peptide was used to target DCs and induce a robust T cell protective response [142, 143].

### Viral infections and mRNA vaccines

A great amount of work has been dedicated to designing vaccines for viral infections following the novel coronavirus disease (COVID-19) pandemic caused by severe acute respiratory syndrome coronavirus 2 (SARS-CoV-2), specifically in the form of IVT mRNA vaccines. While the previously described subunit vaccines utilized a fragment of the target antigen in order to elicit an immune response, mRNA vaccines function differently to produce a similar humoral response. Specifically, the mRNA for a protein of interest, the spike protein in the case of SARS-CoV-2, is generated and injected into the patient using a nanocarrier. The principle behind IVT mRNA technology is as follows. First, mRNA containing a 5’ cap, 5’ and 3’ untranslated regions, the open reading frame, and a 3’ poly(A) tail is incorporated into a nanocarrier that will facilitate cell membrane interactions and endosomal escape for in vitro translation, these will be discussed in an upcoming section. After entering the cell, a fraction of the exogenous mRNA will escape the endosome and enter the cytoplasm. The mRNA will then undergo translation using the cell’s native machinery. Eventually, exonucleases will degrade the mRNA and terminate the translation process for the protein of interest. The newly expressed proteins undergo  post-translational modifications and are degraded into antigen peptides loaded onto MHC molecules for presentation to immune effector cells. From here, cellular or humoral immunity will arise and provide the patient immunity to the antigen that was processed and presented. In this way, if the patient is exposed to the SARS-CoV-2 virus in the future, existing antibodies are capable of binding to the virus and preventing it from replicating. With this general idea in mind, several strategies have been employed to design optimal mRNA lipid nanoparticles to be utilized as a vaccine regarding their efficacy, toxicity, and stability.

The design of lipid nanoparticles for mRNA vaccines should take into consideration several elements including: i) mRNA target and possible nucleotide modifications to ensure immunogenicity and recognition by RNA sensors, ii) an optimized lipid nanoparticle formulation that can encapsulate and deliver the target mRNA, and iii) long-term storage of the nanoparticle. Other types of nanoparticles, both organic and inorganic, are summarized in Table 2. Nucleotide modifications are crucial when developing mRNA vaccines, as they can otherwise be recognized by RNA sensors, including TLR7 and TLR8, and elicit an unfavorable innate immune response [144]. While the end goal of these vaccines is to activate the immune system, doing so preemptively may drastically reduce mRNA translation and render the treatment ineffective [145]. Standard mRNA modifications that reduce immunogenicity include modifying nucleotides with pseudouridine, 2-thiouridine, N6-methyladenosine, N1-methylpseudouridine, and 5-methylcytidine as they aid in preventing mRNA from activating TLRs, or making the mRNA undetectable by RIG-I and PRK pathways in the cases of pseudouridine and 2-thiouridine [146]. By employing nucleotide modifications, researchers can enhance the stability of mRNA used in these nanoparticle vaccines and, ultimately, increase protein translation.

**Table 2:** Nanoparticle vaccines targeted towards viral antigens

Nanoparticle	Size (diameter)	Antigen	Viral target	References
Gold	20–40 nm	West Nile Virus envelope protein	West Nile Virus	[147]
Gold	12 nm	M2e	Influenza A	[148]
Iron oxide	25 nm	HIV-1 Envelope glycoprotein SOSIP trimers	HIV-1	[149]
Mesoporous silica	120 nm	E2	Bovine viral diahorrea virus	[150]
Chitosan	571.7 nm	Killed swine influenza A virus H1N2	Swine influenza virus (H1N2)	[151]
PLGA	200–300 nm	Inactivated virus H1N2	Swine influenza virus (H1N2)	[152]
PLA/PLGA	474–940 nm	Hepatitis B surface antigen	Hepatitis B virus	[153]
PLGA/lecithin	300 nm, 1 μm, and 3 μm	HPV-L1 Pentamer	Human papillomavirus	[154]
PLGA	972.5 nm	Influenza split vaccine antigen	Influenza H5N1	[155]
MD39-6xHis:Ni–NTA (1:40)	150 nm	BG505 MD39 trimer	HIV	[156]
DOPC:DOPG (4:1) and DMPC:DOPC:DOPG (2:2:1)	64.5 nm, 150 nm, and 203 nm	membrane-proximal external region (MPER) from envelope subunit gp41	HIV	[157]
Virus-like particles	10 nm	HPV16 L1 capsomeres	HPV	[158]
Protein nanoparticle (ferritin)	20–50 nm	HIV-1 envelope and H1 influenza hemagglutinin	HIV and Influenza (H1N1)	[159]

Other approaches to optimizing the mRNA are to i) employ a cap at the 5’ end to eliminate free phosphate groups in the mRNA, which can both increase the stability and translation efficiency by recruiting additional transcriptional machinery, ii) incorporate untranslated regions (UTRs) that regulate transcription, and iii) reduce RNA exonuclease activity with a poly(A) tail on the 3- end. There have been several capping agents that have been employed over the years, including eukaryotic translation initiation factor 4E (eIF4E), anti-reverse cap analogs (ARCA), and CleanCap [146, 160, 161]. In essence, the development of new capping agents was to promote the orientation of the cap on the mRNA, as 20% of eIF4E capped mRNAs were either not capped or capped incorrectly, resulting in poor efficacy. UTRs influence both the translation efficiency and mRNA half-life. 5’ UTRs influence protein expression and should be designed to have a minimal amount of GC content as this can cause complex geometries that may hinder ribosomal recruitment and, therefore, start codon recognition [162]. Similarly, 3’ UTRs should be kept to an optimal length as longer sequences decrease the half-life of the mRNA [163]. Finally, the poly(A) tail promotes mRNA transcription by binding poly(A)-binding proteins (PABP) that recruit eIF4E and eIF4G on the 5’ cap to promote a circular mRNA structure that diminishes immunogenicity [164].

**Fig. 8.**
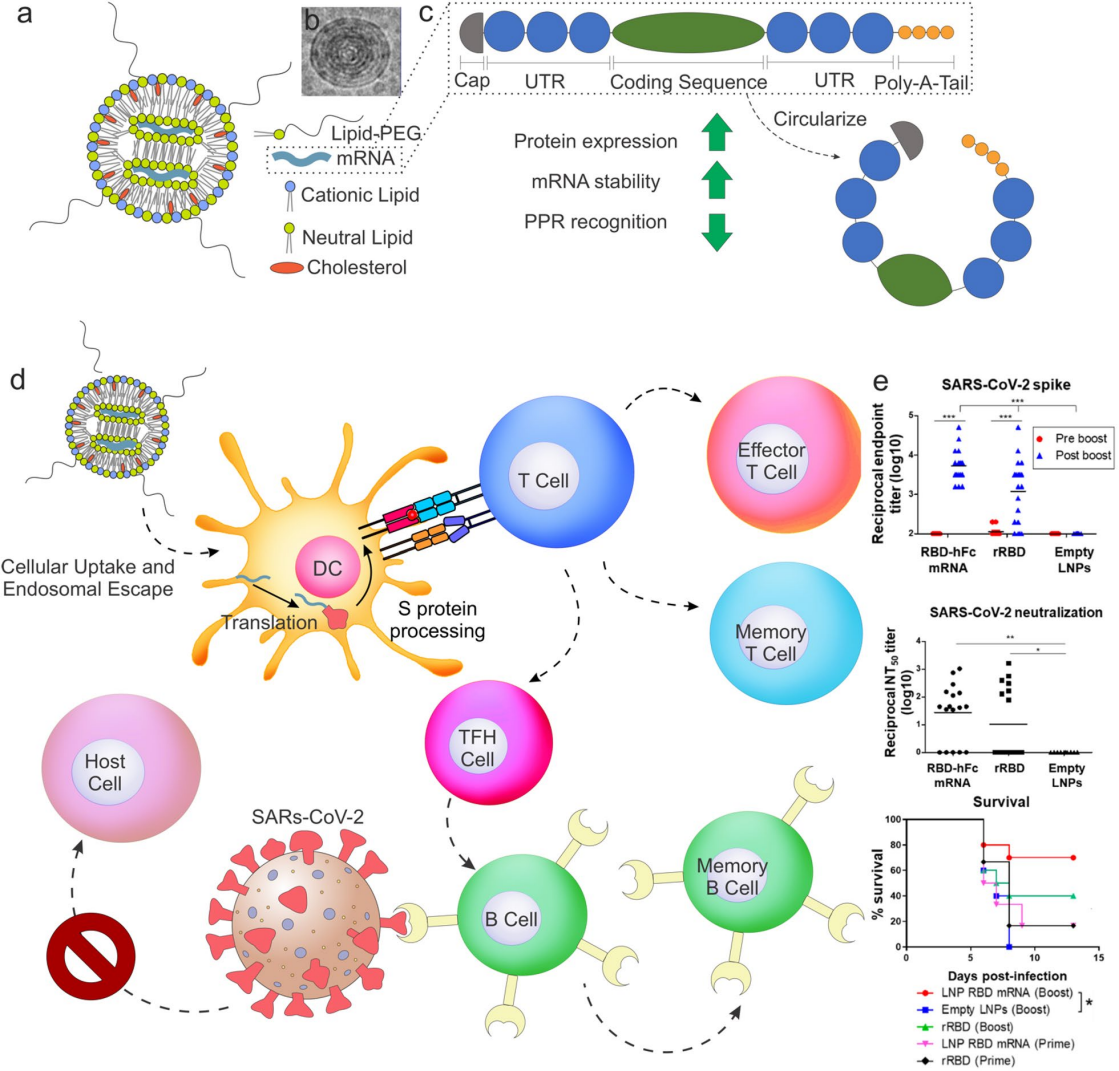
mRNA modifications and delivery for viral vaccines. **a** General lipid nanoparticle formulations for mRNA delivery, peg-ylated lipids improve circulation while cholesterol and cationic lipids provide structural stability and facilitate endosomal escape and cellular uptake, respectively. **b** Cryo-EM image of a lipid nanoparticle. **c** Commonly utilized mRNA modifications that increase the efficacy of the delivered mRNA by increasing protein expression and mRNA stability while mitigating immune activation via pattern recognition receptors. **d** Mechanism of action for viral vaccines. Following delivery and cellular uptake of the mRNA encapsulated lipid nanoparticles , the transcript is able to escape the endosome and undergo translation, in this schematic the mRNA codes for the spike protein. T he spike protein is then processed by proteases and presented to T cells in lymph nodes via the MHC-II and TCR complex. Next, T cells begin to proliferate and differentiate into effector T cells and T follicle helper cells that can stimulate B cells to produce antibodies specific o the spike protein antigen. Following future exposure to SARS-CoV2, the patient’s immune system will be primed and capable of combating the virus. **e** Serum samples collected 23 days (“preboost”) and 46 days (“postboost”) from hACE2 transgenic mice treated following initial priming. Lipid nanoparticle  RBD-hFc mRNA treated mice display elevated SARS-CoV-2 spike-specific IgG antibodies (**e1**) and neutralizing antibodies (**e2**) following the booster compared to mRNA delivered without a lipid nanoparticle  and lipid nanoparticles without the target mRNA. **f** Survival of mice groups following treatment shows that boosted mice had a higher survival rate. **g** Weight of treated mice returned normal two weeks post booster for mice treated with lipid nanoparticle  RBD-hFc mRNA. Untreated mice did not survive past day 7. Reproduced with permission from ref [173, 174].

Two of the most prominent mRNA vaccines currently available are for the recent novel coronavirus disease caused by SARS-CoV-2, specifically Pfizer/BioNTech’s BNT162b2 and Moderna’s mRNA-1273 vaccines. Recently, the immunopathogenesis of COVID-19 has been extensively reviewed [165]. The spike protein is a class I fusion glycoprotein that has been identified as the major surface receptor on the coronavirus and is the primary target for generating neutralizing antibodies. However, producing viable cell lines with clinical-grade spike proteins can be an arduous and time-consuming task, which is detrimental to developing a vaccine quickly and efficiently. To this end, researchers at both of these pharmaceutical companies decided to leverage an mRNA vaccine that elicits both cellular and humoral immunity with a notably decreased manufacturing time [166, 167]. Furthermore, it was previously demonstrated that modifying uridine in mRNA, via a N1-methylpseudouridine modification, allows for mRNA vaccines to avoid endosomal TLR-mediated microbial-associated molecular pattern (MAMP) activation and prevents inciting an innate immune response [168]. Thus, the mRNA-1273 Moderna vaccine utilizes a N1-methylpseudouridine modification on their optimized spike protein mRNA [SARS-CoV-2S(2P)] in addition to 5’ and 3’ UTRs and a 3’ poly(A) tail (Fig. 8c) [169]. When designing the lipid nanoparticle to deliver mRNA-1273, Moderna chose a ratio of SM-102: 1,2-distearoyl-sn-glycero-3 phosphocholine (DSPC): cholesterol (ionizable cationic lipid: neutral lipid: cholesterol: (heptadecan-9-yl 8-((2-hydroxyethyl) (6-oxo-6-(undecyloxy) hexyl) amino) octanoate} PEG2000-DMG (monomethoxypolyethyleneglycol-2,3-dimyristylglycerol) 50:10:38.5:1.5) with a known pKa of 6.75 [170, 171]. The pKa of these ionizable lipids falls within the optimal 6.6–6.9 range described previously for eliciting an adaptive immune response following mRNA vaccination. Furthermore, the small amount of PEG-ylated lipids used is consistent with previous reports used to control the lipid nanoparticles size, prevent mRNA leakage, and increase stability. Pfizer-BioNTech utilized the same mRNA modifications with a different lipid composition and molar ratios for their BNT162b2 mRNA vaccine containing the same 4 major components, (4-hydroxybutyl) azanediyl)bis(hexane-6,1-diyl)bis(2-hexyldecanoate) (an ionizable lipid), 2-[(polyethylene glycol)-2000]-N,Nditetradecylacetamide, DSPC, cholesterol. Unlike Moderna’s lipid nanoparticle, Pfizer’s has a pKa of 6.09 that is below the cited optimal range for eliciting an mRNA vaccine adaptive immune response [171, 172].

## Promoting tolerance for autoimmunity and suppressing chromic inflammation

Autoimmune diseases are marked by abnormally low activity or overactivity of the patient’s immune system, which limits the body’s ability to combat foreign invaders or causes the body to lose tolerance towards native antigens, and develop autoantibodies that recognize their own tissue as foreign, respectively [175]. According to the American Autoimmune Related Disease Association (AARDA) approximately 50 million Americans suffer from autoimmune disorders (https://aarda.org/). However, the factors that contribute to autoimmune diseases are immense and complex as they can combine factors from both innate and adaptive immunity. In pathological autoimmune disorders innate immune components, such as pattern-recognition receptors (PRRs), such as TLRs, are stimulated and activate myeloid cells, innate-lymphoid cells, and various populations of T cells that ultimately contribute to the disease pathology. However, when the adaptive immune system is responsible for the disease, B and T cells increase the range of antigens they can recognize but, in doing so, lose the ability to distinguish between exogenous and self-antigens. Affecting the lining of synovial tissue in diarthrodial joints, the lining of the intestines, nervous tissue, and systemically throughout the body are some of the manifestations of autoimmune diseases. These include rheumatoid arthritis (RA), Inflammatory Bowel Disease (IBD), multiple sclerosis (MS), and systemic lupus erythematosus (SLE), to name a few [176–179].

Traditionally, treatment of autoimmune diseases has centered around suppressing the autoimmune response of the disorder by delivering immunosuppressants or other therapeutic agents that interfere with cell activation or migration via systemic or locally targeted methods [180, 181]. However, while some disease-modifying anti-rheumatological drugs (DMARDs) are very potent and efficient in providing symptomatic relief and slowing the progression of the disease, they do so at the risk of moderate to severe off-target effects that may result in toxicity. For instance, calcineurin inhibitors, such as cyclosporine, are used to treat RA by blocking T cell activation and IL-2 production, which is associated with T cell proliferation, but may also confer nephrotoxicity concerns to the treated patient [182]. A rise in the number of biologics for the treatment of autoimmune diseases has occurred in response to the associated health issues created by the off-target effects of DMARDs. These biologics are intended to ameliorate these health concerns. Compared to traditional drugs, biological drugs tend to be antibodies, interferons, fusion proteins, or synthetic proteins that either block the effects of pro-inflammatory cytokine or act on immune-competent cells, including T cell and B cell targeted biologics. One example is the biologic Tocilizumab, a monoclonal antibody, that is an IL-6 receptor antagonist responsible for inhibiting the pro-inflammatory cytokine signaling of IL-6 in RA patients and is intravenously injected [183]. While biologics are generally safer than DMARDs and can be used in conjunction to aid in the treatment of patients suffering from autoimmune diseases, there exist several limitations that still remain to be addressed including: i) delivery to the target cells, ii) degradation and clearance in vivo, and iii) transport across biological barriers. To this end, there have been various reports of utilizing nanotechnology to address the aforementioned limitations.

One promising application of nanotechnology in treating autoimmune diseases has been observed in patients suffering from MS. Multiple sclerosis (MS) is an autoimmune disease that affects the central nervous system (CNS), causing patients to have deficits in neurocognitive ability, sensation, motor, and autonomic functions as a result of the activation of CD4^+^ autoreactive T cells and a cascade of cellular events that eventually leads to chronic inflammation and destruction of the myelin sheath by myelin-reactive auto-T cells [184]. In short, immune cells, such as microglia, that reside in the CNS begin to upregulate MHC-I and -II, cell surface costimulatory molecules, and cytokines that allow CD4^+^ and CD8^+^ T cells, monocytes, macrophages, and DCs to enter into the CNS, via the blood–brain barrier (BBB), following their activation [185]. Of these newly migrated cells, myelin-reactive auto-T cells initiate an inflammatory cascade and cause the demyelination of neurons, resulting in axonal damage and loss of neuronal function. The demyelination processes provide a positive feedback loop to further promote the inflammatory process while simultaneously causing damage to the BBB, stimulating oxidative and nitrosative stress pathways, and activating macrophages [186]. While there is no available treatment to regenerate myelin following its destruction, nanotechnology has been utilized in MS to deliver therapeutic agents across the BBB in a targeted manner to alleviate symptoms of inflammation.

### Immunotherapy via drug delivery

Using lipid nanocarriers, one approach has been to deliver therapeutic molecules through the BBB by entering the post-capillary venules. Lipid nanocarriers can encapsulate both hydrophilic and hydrophobic molecules, pass through the BBB by entering post-capillary venules, and have their surfaces engineered to interact with the BBB or specific cells of interest [187]. In 2019, an intranasal lipid nanocarrier was developed to deliver the selective dihydroorotate dehydrogenase inhibitor Teriflunomide (TFM) [188]. TFM has been demonstrated to have a cytostatic effect on proliferating B cells and T cells as well as activated astrocytes and microglia by preventing the synthesis of various cytokines and interfering with the interaction between T cells and DCs [189]. Normally, therapeutic molecules have difficulty penetrating the BBB due to this barrier’s innate hydrophilicity and can also cause hepatotoxicity if delivered systemically. However, the authors could circumvent these limitations by loading TFM into a lipid nanocarrier. Moreover, by adopting an intranasal delivery method, the lipid nanocarriers can avoid hepatic clearance and toxicity and also have a large surface area of vascular tissue to permeate through to increase the number of particles reaching their target location. Due to their small size (~ 100 nm) and composition (i.e., combining HPMC K4M and poloxamer 407) these lipid nanocarriers allow for biodegradable, biocompatible, and mucoadhesive systems.

Another noteworthy study used solid lipid nanoparticles (SLNs) to administer the polyamine methylthioadenosine orally (MTA). In this study, SLNs were synthesized by a microencapsulation technique where steric acid, MTA, Tween 80, and phospholipid 90 G (PL) were combined and mixed isothermally to generate approximately 100 nm SLNs with a negative zeta potential (~ -8 mV). It has previously been described that oral delivery of this SLN system can promote the delivery of several drugs to the CNS [190, 191]. In order to mimic MS, a mouse model was utilized where the animal was subjected to the copper chelating agent cuprizone in order to induce demyelination. Pharmacokinetically, it was found that by incorporating MTA into a SLN the researchers were able to enhance the concentration of the compound that was being delivered at various time points across 4 h. Moreover, the circulation time was nearly tripled using the SLN, where the elimination half-life of the compound was 28 min when administered alone versus 1.25 h when delivered using the lipid platform. Following demyelination, the mice experienced decreased locomotor activity; however, measurements obtained from actophotometer and Rotarod tests demonstrated that the mice treated with MTA loaded in the SLN were able to improve their locomotor activity to 71% relative to the cuprizone-only group, whereas the MTA-only group improved locomotor active by 49%. Similarly, the rotarod test demonstrated an increase in overall coordination, grip strength, and balance of the animals by 95% in the MTA-SLN condition relative to controls and a 68% increase in the MTA-only condition. A histopathological study revealed that the cuprizone-only group had average myelination of 57%, whereas the MTA and MTA-SNL conditions had 65% and 80% myelination, respectively, after 30 days of treatment. Overall, this study shows the advantages of utilizing nanotechnology in the effective delivery and treatment of MS.

Rheumatoid arthritis (RA) has been another area of interest for delivering therapeutic drugs utilizing nanotechnology. RA is a chronic and systemic autoimmune disease that predominantly affects the synovial joints, where it is characterized by inflammation of the synovium that can lead to cartilage and bone erosion, and can lead to progressive disability and premature death as there are risks associated with extra-articular symptoms such as keratitis, pericarditis, and pulmonary granulomas [192]. One key contributor to the pathogenesis of RA and other autoimmune diseases is Th17 cells. Th17 cells are known to express pro-inflammatory cytokines (i.e., IL-17[A-E], IL-6, IL-21, IL-22, and TNF-α), and their differentiation is promoted by exposure to a series of cytokines (TGF-β, IL-6, and IL-21) that activate STAT3 and induce the expression of lineage-specific transcription factors RORγt and RORα [193, 194]. Of the aforementioned cytokines produced by Th17 cells, IL-17A contributes to RA pathology by stimulating fibroblast-like synoviocytes (FLS), causing osteoclasts to mature, and recruiting other immune cells, such as neutrophils, macrophages, and B cells [195]. While usually responsible for lubricating the joints by producing hyaluronic acid and lubricin, once activated, FLS matures and begins heavily expressing TLR1-6 due to an increase in TNF-α and IL-1β [196, 197]. The overexpression of toll-like receptors (i.e., TLR3) and secreting factors like IL-6 in conjunction with cell–cell interactions and type I interferons ultimately leads to enhanced B cell differentiation, which leads to a variety of autoantibodies, T cell, and macrophage activation, which can promote inflammation [176, 198]. Moreover, activated FLS can begin destroying the extracellular matrix and surrounding cartilage by overproducing matrix metalloproteinases (MMPs) to promote a pro-inflammatory environment further.

**Fig. 9:**
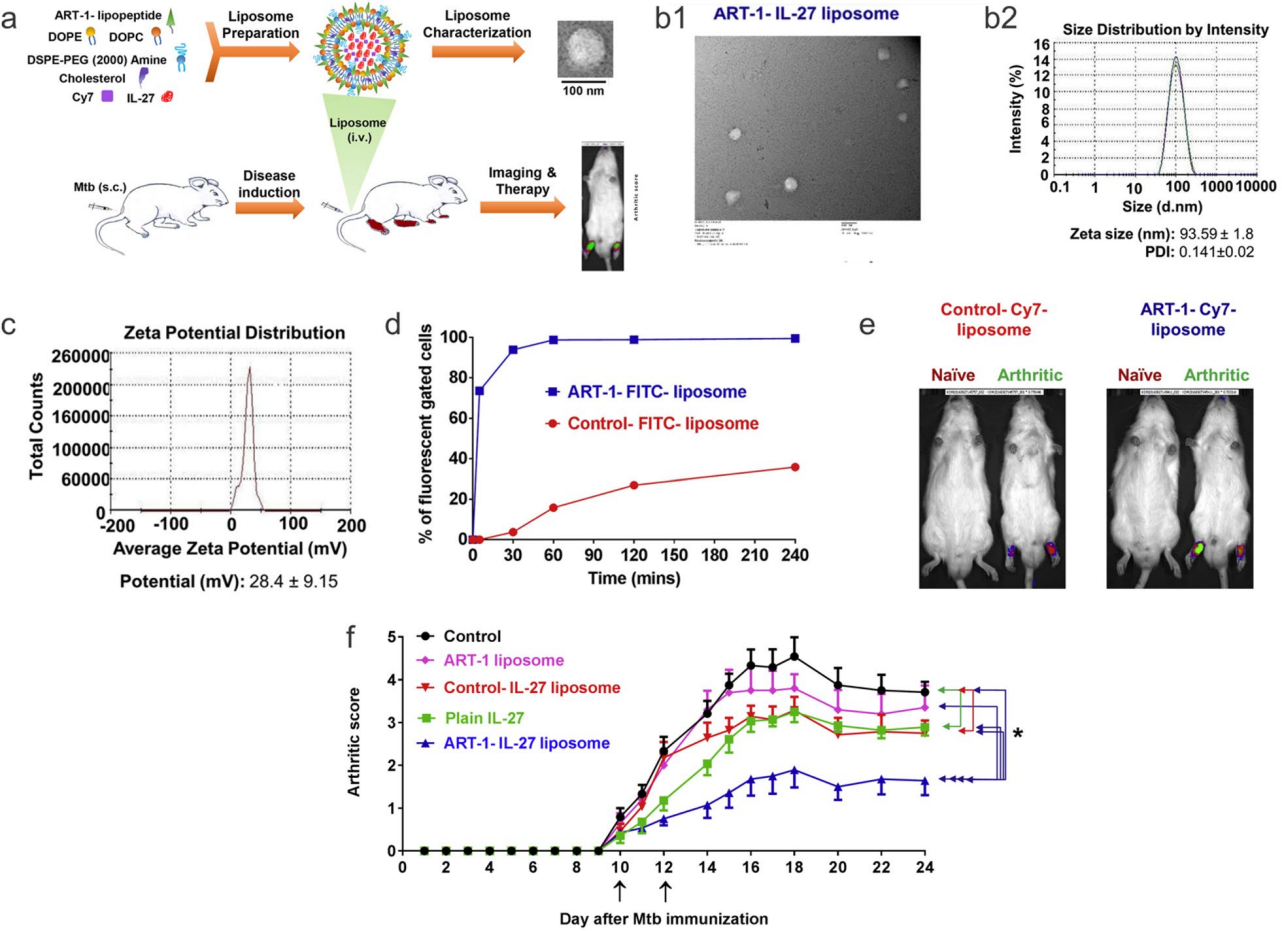
Peptide targeted delivery of liposomes to modulate cytokine release in arthritis. **a** Schematic highlighting liposome components and in vivo delivery to arthritic rats. **b** Material characterization of ART-1-IL-27-liposomes. **b1** and **b2** illustrate the uniformity and monodispersity of the ART-1 targeted liposome by TEM and DLS respectively. **c** Zeta potential measurement to highlight positive induced charge following ART-1 incorporation. **d** Flow cytometry data corresponding to cellular uptake of ART-1-FITC-liposomes and Control-FITC-liposomes at 250 nM concentrations at various time points. Liposomes modified with ART-1 are capable of targeting and have better uptake efficiency than the control liposomes in vitro. **e** Real-time fluorescent imaging of naïve and arthritic rats 4 h post I.V. injection. There is increased accumulation in the arthritic joints of the rats with the ART-1 targeting ligand relative to the control. Naïve rats do not demonstrate particle accumulation. **f** Arthritic score of groups of rats treated with combinations of the ART-1-IL-27 liposome. The group that received with ART-1-IL-27-lipsosme demonstrate better recovery with a lower arthritic score than the control and groups treated with either ART-1 liposome, Control-IL-27 liposome, or Plain IL-27. Reproduced with permission from ref [200]

To this end, a liposome drug delivery system was designed using a novel peptide (ART-1) to preferentially target inflamed cells in joints. This platform also encapsulated IL-27 as a therapeutic agent as it has been demonstrated to inhibit Th17 differentiation while also modulating angiogenesis, osteoclastogenesis, and matrix degradation that are associated with tissue damage and inflammation [199, 200]. The liposome was formulated with DOPC (1, 2-Dioleoyl-sn-glycero-3-phosphocholine), DOPE (1, 2-Dioleoyl-sn-glycero-3-phos-phoethanolamine), cholesterol, and DSPE-PEG (2000) amine (1, 2-distearoyl-sn-glycero-3-phosphoethanolamine-N- [amino (polyethyleneglycol)-2000] and the ART-1 lipopeptide was custom synthesized and incorporated in the formulations, with a size range from 53 nm-165 nm. When this platform was delivered to the animal model, it was determined that IL-27 containing liposomes without the targeting peptide failed to inhibit disease progression to the same extent the targeted liposomes were able to. Furthermore, by directing the liposome containing the therapeutic molecules to inflamed joints, systemic exposure to the cytokine was minimized, as were off-target responses (Fig. 9).

Another critical component that contributes to the pathology of RA is activated macrophages. Once activated by the cascade of events described above, macrophages can also release pro-inflammatory cytokines (i.e., IL-1, IL-6, and TNF-α), digestive enzymes, and proteases such as collagenases and MMPs, and produce ROS that can further damage healthy tissues. To this end, nanotechnology has been implemented to deliver therapeutic agents to combat several of these deleterious pathways observed in RA. When macrophages are subjected to prolonged inflammation, they begin to express different markers than would be observed in a healthy state. It has been known that of these receptors, folate receptor-β (FRβ) is selectively elevated in RA synovial macrophages [201]. Thus, folate-mediated targeting, specific for FRβ, has been one approach utilized to specifically target activated synovial macrophages. For instance, a study performed in 2015 was able to develop a liposome functionalized with FRβ that can encapsulate the DMARD methotrexate (MTX) to decrease the harmful side effects and improve the drug's delivery efficiency [202]. In this study, the liposome was composed of DOPE, cholesterol, and N-(carbonyl methoxypolyethylene glycol-2000)- 1,2-distearoyl-sn-glycero-3-phosphoethanolamine (DSPE-MPEG) and functionalized with a folic acid peptide specific for FRβ in order to encapsulate MTX and precisely deliver the drug to synovial macrophages. A collagen-induced mouse arthritis model was utilized, and it was found that the formulation used was able to reduce expression of both CD39 and CD73, the expression of which is correlated with FRβ expression levels, in joint-infiltrating macrophages. By specifically targeting this population of macrophages, the authors could reduce off-target effects and observe a more favorable clinical score for the targeted system than the bare liposome or MTX treated conditions. This group also utilized the same platform to deliver siRNA for myeloid cell leukaemia-1 (*MCL1*) to induce apoptosis in synovial macrophages [203].

While inflammatory processes are necessary to initiate healing processes, chronic inflammation, as is observed in chronic wounds, can prevent the injury site from returning to a point of homeostasis. Wound healing is a four-phase immune system response following skin damage that aims to neutralize pathogens and regenerate tissue to close the wound. The first part, homeostasis, starts at the time of injury and ends with the arrival and coagulation of platelets. Patelets then enhance the transition to the inflammation phase by excreting TGF-β1 and Platelet-Derived Growth Factors (PDGFs), which attract neutrophils to the injury site. Neutrophils also release antimicrobial substances to counter bacterial pathogens, and cytokines that recruit macrophages to the injury site. M1 macrophages are responsible for removing cellular debris and apoptotic cells. In the third phase, the proliferation of keratinocytes, fibroblasts, and epithelial cells regenerates skin tissue. The wound healing process turns to an anti-inflammatory state by releasing TGF-β1 and cytokines, and repolarizing macrophages to an M2 state. The release of IFN-γ by macrophages favors fibrogenesis. The final phase of the process sees little cell proliferation and ECM remodeling but rather a decrease of immune cell activity. Macrophages and fibroblasts undergo apoptosis or exit the injury site [204]. For patients suffering from chronic wounds, the wound healing process is unable to progress from the inflammatory stage and tissue remodeling does not occur.

Nanoparticles can enhance tissue regeneration by repolarizing macrophages, before the injury site shifts into the third anti-inflammatory phase. Macrophages then secrete anti-inflammatory biological substances, which help drive the injury site to an anti-inflammation state. One-way nanoparticles can do this is by delivering small molecules. Xia et al. loaded Rebamipide onto chitosan nanoparticles that inhibit the NF-kB signaling pathway, preventing polarization into the M1 phenotype [205]. The small molecule can also be in the form of a sugar unit. Dong et al. delivered galactose-α-1,3-galactose (α-gal) in liposomes to mice suffering from splinted wounds. α-gal showed to accelerate wound healing and closure by enhancing macrophage invasion and lowering the M1 to M2 ratio [206]. In addition, the small molecule does not necessarily have to dissociate from the nanoparticle. Silica nanoparticles covered in mannose cluster the mannose receptor on the macrophage and polarize the cell into M2. The authors found higher levels of fibroblast proliferation [207].

### Immunotherapy via biologics

Alternatively, new research has been conducted on developing immunological tolerance with DCs that have immune-suppressive capabilities against a specific antigen. Rather than trying to modulate cellular signaling pathways to reduce inflammatory signals or prevent cells from overproducing antibodies and/or migrating to the disease site, this approach takes advantage of existing cellular functions of the immune system to alleviate autoreactive lymphocytes that are responsible for the disease. Specifically, DCs are highly phagocytic immune surveillance cells that recognize a plethora of pathogen-associated molecular patterns (PAMPs) and damage-associated molecular patterns (DAMPs) via PRRs and TLRs organized on their outer membrane. By internalizing potential foreign material, DCs can initiate and regulate functions observed with adaptive immunity by presenting antigens via the MHC-II complex to T cells to stimulate their differentiation. In addition to stimulating T cell differentiation, DCs also tightly regulate T cell development by both central and peripheral tolerance. During central tolerance, DCs present self-antigens to T cells located in the thymus and induce apoptosis when they respond strongly. A similar phenomenon is observed in lymph nodes for peripheral tolerance checks to maintain homeostasis and prevent autoimmune responses [208, 209].

Traditionally, a combination of pharmacological agents, including rapamycin or dexamethasone, cytokines (e.g., TGF-β, TNF-α, IL-1β and/or IL-10), and a variety of the autoantibody desired to generate tolerance towards are introduced to ex vivo purified DCs to generate tolerogenic DCs (tolDCs) [210]. By tuning the chemical cocktail used to induce tolDCs, they can exhibit various phenotypes, including differences in migratory behavior, cytokine release, and type of tolerogenic outcome induced in T cells. After generating the tolDCs with the desired phenotype, the cells can then be transplanted back into the patient to diminish the cellular autoimmune responses via peripheral tolerance mechanisms to cause T cell anergy, deletion, or conversion to a Treg phenotype. While this approach has proven promising in clinical trials, there are several limitations that need to be overcome including: i) the isolation, purification, and culturing conditions of primary DCs requires careful technical experience and ii) generating tolDCs has to be performed on a per-patient basis as there is a risk of histocompatibility complications [211]. However, by implementing nanotechnology and functionalizing nanoparticles with antibodies present on DCs, including CD205, CD206, CD40, or CD11c, various groups have been able to formulate nanoparticle vaccines using tolDCs to modulate cellular responses in a targeted manner [212–215].

One example of generating tolDCs can be observed in the treatment of MS. During this study, several potential autoantigens were identified that play an active role in the demyelination, BBB penetrating, and inflammatory processes, including oligodendrocyte-associated proteins such as myelin basic protein (MBP) and myelin oligodendrocyte glycoprotein (MOG). After MBP was chosen as their target to promote tolerance, small unilamellar vesicles (SUVs) were synthesized via a solid-phase technique and were fabricated from egg phosphatidylcholine (PC) and monomannosyl dioleyl glycerol (ManDOG), which served as a targeting agent towards mannose receptors. These SUVs were then mixed with the MBP peptides to encapsulate them. Controls without the peptide and without the mannose targeting agent were also synthesized similarly but without the specified component. DCs were isolated from the rat’s blood that was utilized in the study for ex vivo data, and the liposome formulation was also delivered systemically to elucidate the in vivo targetability and efficacy. Ultimately, it was discovered that exposure to mannose residues on the surface of liposome carriers is necessary to facilitate delivery to DCs as the nontargeted liposome with the same peptides loaded were significantly less efficient [216]. This was observed by noting the mannosylated liposome’s ability to promote anti-inflammatory, neuroprotective, and regenerative effects. Following treatment, there was a twofold decrease in the concentration of circulating antibodies for MBP, implying systemic suppression of autoreactive B cells, a significant downregulation of IL-2 and IFN-γ, and enhanced expression of BDNF, which is known to induce remyelination [217]. A similar study from 2015 utilized biodegradable polymeric nanoparticles composed of poly(lactic-coglycolic acid) (PLGA) that were 400-500 nm to deliver a MBP peptide [218]. Similar to the previous study, the authors were able to induce potent T cell tolerance to MBPs, reduce immune cell infiltration, and limit cytokine production by protecting their MPN that was conjugated to the surface of the polymeric particle and delivered via IV infusion.

Another promising technology for the delivery of biologics is cell-derived exosomes. Exosomes derived from MSCs have been demonstrated to exhibit immune suppressive properties that can aid in regulating the immune system [219]. In the context of RA, MSC-derived exosomes can ameliorate symptoms such as cartilage degradation and have even been found to induce endogenous cartilage repair [220]. Recently, work done by Tavasolian et al. demonstrated that by isolating exosomes from healthy MSCs that overexpress anti-inflammatory miRNA, specifically miR-146a and miR-155, they could significantly alter Treg populations in collagen-induced arthritis mice. The control over Tregs was observed in conjunction with an increase in anti-inflammatory cytokines, such as IL-10 and TGF-β, and demonstrated that these microRNA transduced exosomes could elicit a therapeutic effect on the immune system CIA-mice [221]. Similar studies were performed by Meng et al. and Zheng et al. The former utilized MSC-derived exosomes carrying miR-320a to regulate RA-FLSs in patient derived samples and was able to attenuate arthritis and bone damage in vivo in mice with CIA by suppressing RA-FLS’s ability to activate, migrate, and invade [222]. Meanwhile, the latter utilized miR-192-5p to downregulate expression of RAC2, which is commonly upregulated in RA synovium and macrophages and can lead to an overproduction of nitric oxide and an inflamed synovium. Using CIA rats, the authors were able to show a decrease in the amount of proinflammatory cytokines (i.e., IL-1β and TNF-α) produced and a reduction in nitric oxide accumulating in the serum [223].

Nanoparticles have also been utilized to deliver biologics to induce macrophage polarization for accelerated woundhealing. The delivery of biological substances also shifts the macrophage into the wound healing state. Xiao et al. loaded a Fibroblast Growth Factor (bFGF) onto an iron oxide nanoparticle. Sustained release of bFGF over 10 days can polarize the macrophage to the M2 state. In vivo experiments showed accelerated wound healing through increased muscle thickness over the control [224]. In addition to growth factor delivery, nucleic acid delivery can also repolarize macrophages. Whitehead et al. delivered TNF-α siRNA in a lipidoid nanoparticle to macrophages. and downregulates MCP-1 while reducing the number of macrophages entering the wound in the M1 state. Lower levels of M1 macrophages lead to rapid wound healing [225].

Nanoparticles can also deliver biologics for heart tissue regeneration. The heart tissue regeneration process is similar to that of wound tissue regeneration, and the stimuli for repolarization are the same [226]. Conjugating IL-4 to the AuNP leads to the secretion of anti-inflammatory cytokines that shift the polarization of the macrophage into the M2 state. The result was functional heart muscle regeneration [227]. Delivery of CD146 and IGF1 shows the higher phagocytic activity of M2 macrophages in systems undergoing myogenesis [228]. Gold-silver nanoparticles promote MHC protein expression, upregulate the expression of myogenic transcription factors, while also activating the p38 MAPK signaling pathway. The Au-AgNPs was able to thicken the skeletal muscle in an in vivo model [229]. On the other side of the spectrum, Lee et al. silenced the transcription factor IRF5 via delivery of RNAi in a lipid nanoparticle. Suppression of the IRF5 gene leads to higher M2 macrophage cell count, faster wound regeneration, and enhanced infarct healing [230].

## Summary and future perspectives

Nano-immunoengineering provides a powerful and versatile way for scientists and engineers to control and interrogate immune cell functions. One of the biggest appeals of these nanoparticles is that they can serve as delivery vehicles for in situ cell programming, allowing for cell immunization (e.g., delivering nucleic acids to DCs) or cell engineering (CAR T-cells) in vivo. Such a feat is difficult using current viral vector technology due to the smaller packaging capacity of viruses. This is especially important considering the future generations of CAR that incorporate additional co-stimulatory domains or dimerization domains for enhanced anti-tumor immunity or safety, where the constructs can exceed the cloning capacity of viral vectors. Synthetic nanomaterials can overcome such limitation and enable the delivery of these intricate constructs. The pre-existing immunity in the population for adeno-associated virus (AAV)-based therapies or immunogenicity and tumorigenicity for lentivirus-based therapies further preclude the wide use of viral vector technology. On the other hand, nano-immunoengineering of nonviral vectors can be easily tuned to have gain of function modalities, such as PEG engineering to increase colloidal and serum stability, antibody conjugation to improve targeting, and nuclear localization signal to improve gene transfer efficiency. The latter is especially critical, as one major obstacle concerning the delivery of nucleic acid cargoes for nano-immunoengineering, particularly DNA, is the low gene transfer efficiency. We envision that increasing the delivery of pDNA will drastically increase the overall effectiveness of the in-situ engineering applications and hence a better therapeutic outcome. This allows for scaled-up manufacturing of the therapeutic products and decreased overall costs and preparation time.

Through proper selection of the materials, researchers can better control the safety profile of the systems or tune the property of the nanomaterials for controlled and sustained release. For instance, inorganic nanoparticles such as iron oxide can be used as MR contrast agents for tracking immune cells, or UCNP as light transducers for controlling synthetic protein activities to limit off-target toxicities. In some cases, such as cancer vaccines, the delivery material themselves (e.g., cationic lipids and polymers) could serve as adjuvants to enhance the immune response. Conversely, the degradation of PLGA (and the release of lactic acid) can dampen the local immune microenvironment and provide immunomodulatory effects. Therefore, selecting the optimal nanomaterials for the specific application requires careful consideration for maximized efficacy. A deeper understanding of the physiochemical properties of these nanomaterials and how they interface with the immune system will allow researchers to address underlying challenges in current therapies, as well as explore new frontiers in treatment. In essence, nano-immunoengineering has the potential to effectively counteract disease progression in a cost-effective manner, while still being able to maintain safety and therapeutic efficacy, hence enabling a wider population to be benefited from the next-generation immunotherapies.

## Data Availability

Not applicable.
